# Millimeter-Scale Single-Crystal α‑MoO_3_ Nanosheets Grown by Alkali Salt-Assisted Chemical Vapor Deposition

**DOI:** 10.1021/acsnano.6c07396

**Published:** 2026-07-09

**Authors:** Ryan W. Spangler, Thiago S. Arnaud, Caleb Whittier, Bhaveshkumar Kamaliya, Sai S. Tripathy, Patrick E. Hopkins, Elizabeth C. Dickey, Joshua D. Caldwell, Nabil D. Bassim, Jon-Paul Maria

**Affiliations:** † Department of Materials Science and Engineering, The Pennsylvania State University, University Park, Pennsylvania 16802, United States; ‡ Interdisciplinary Materials Science Program, 5718 Vanderbilt University, Nashville, Tennessee 37240, United States; § Department of Materials Science and Engineering, 3710 McMaster University, Hamilton, Ontario L8S 4L7, Canada; ∥ Department of Materials Science and Engineering, 122584 Carnegie Mellon University, Pittsburgh, Pennsylvania 15213, United States; ⊥ Department of Mechanical and Aerospace Engineering, University of Virginia, Charlottesville, Virginia 22904, United States; # Department of Materials Science and Engineering, University of Virginia, Charlottesville, Virginia 22904, United States; ∇ Department of Physics, University of Virginia, Charlottesville, Virginia 22904, United States; ○ Department of Mechanical Engineering, Vanderbilt University, Nashville, Tennessee 37235, United States; ◆ Canadian Centre for Electron Microscopy, Hamilton, Ontario L8S 4M1, Canada

**Keywords:** single-crystal growth, vapor−liquid−solid, van der Waals materials, α-MoO_3_, phonon polaritons, near-field optical microscopy

## Abstract

The orthorhombic van der Waals (vdW) layered crystal α-MoO_3_ is a promising material for infrared nanophotonics; in particular, it may host highly confined hyperbolic phonon polaritons (HPhPs) with wavelength-dependent in-plane anisotropy. However, large-area and uniform single crystals are challenging to grow on substrates, as current α-MoO_3_ growth methods struggle to manage adverse tendencies in size, texturing, and roughness. In this work, we establish an alkali salt-assisted chemical vapor deposition (SA-CVD) growth technique to produce smooth, high-quality, and millimeter-scale single-crystal α-MoO_3_ nanosheets directly on A-plane sapphire substrates. By cosublimating a NaCl source along with α-MoO_3_ during growth, we overcome the size and morphology challenges typical of alkali-free deposition, achieving ultrasmooth crystals with lateral dimensions reaching 6 mm and thicknesses ranging from <6 to 480 nm. We attribute the improved morphology to a molten Na_2_O–MoO_3_ intermediate, which forms on the substrate surface and induces a self-expanding vapor–liquid–solid (VLS) growth mode. The as-grown single-crystal nanosheets exhibit high crystal and optical quality without evident degradation by residual Na, enabling characteristically high HPhP quality (Q) factors (12–30) and long lifetimes (2.7–7.7 ps) as measured by scattering-type scanning near-field optical microscopy (s-SNOM). We relocate the large-area crystals onto arbitrary substrates using a water-assisted layer transfer technique, which effectively removes Na-containing residue and relieves residual strain. This work unlocks millimeter-scale, high-quality, uniform α-MoO_3_ single-crystal growth directly on substrates for large-area implementation in fields including mid-infrared nanophotonics and layered vdW heterostructures.

## Graphical abstract

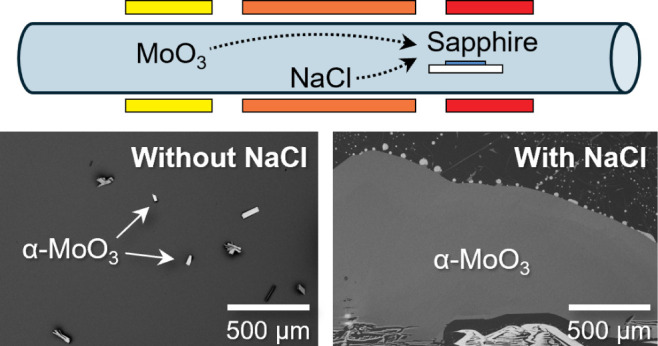

## Introduction

The large-area growth of thin van der Waals (vdW) layered single crystals is instrumental for exploring and implementing high-quality mono- and multilayer materials including graphene,^1,2^ hBN,^3^ transition metal dichalcogenides (TMDs),^4,5^ and others. Furthermore, technologies hinging on the in-plane anisotropy of low-symmetry layered crystals such as SnSe,^6^ black phosphorus (BP),^7^ and certain transition metal oxides such as α-V_2_O_5_ ^8^ and α-MoO_3_ ^9,10^ are particularly reliant on single-crystal morphologies. In many cases, however, large-area, single-crystal growth directly on substrates is challenging and requires further development before widespread adoption can occur.

The α-MoO_3_ crystal structure is composed of distorted Mo–O_6_ octahedra that are strongly and anisotropically bonded in the *a*-*c* plane and ordered into vdW bilayers along the *b*-axis. The orthorhombic unit cell (space group *Pbnm*) has the lattice parameters *a* = 3.962 Å, *b* = 13.860 Å, and *c* = 3.697 Å.^11^ The biaxial anisotropy has recently provoked excitement following the discovery of both in-plane and out-of-plane hyperbolicity in α-MoO_3_,^9,10^ phenomena occurring when two real components of the dielectric permittivity tensor exhibit opposite signs.^12^ These negative permittivity regions are accompanied by the low-loss, tunable, and in-plane anisotropic (elliptic or hyperbolic) propagation of strongly confined hyperbolic phonon polaritons (HPhPs)—infrared (IR) light coupled with polar lattice vibrations—which has spotlighted α-MoO_3_ as a fertile platform for confining and manipulating mid-IR light at the nanoscale.^9,10,13^ As a result, considerable research has explored HPhPs in α-MoO_3_ for applications including directional energy transfer and tunable flat optics.^14–17^ In addition, α-MoO_3_ possesses a high dielectric constant (up to 40),^18–20^ high work function (6.6 eV),^21^ and relatively large band gap (∼3 eV),^22^ making it attractive as a building block for various electronic and optical devices including vdW heterostructures.^23,24^ However, implementation has been hindered by the lack of large-area, uniform growth techniques for single-crystal α-MoO_3_ on substrates.

Commonly, α-MoO_3_ single crystals are prepared as bulk crystals grown using physical vapor transport (PVT)^25,26^ and then mechanically exfoliated to produce thin and atomically smooth samples. However, exfoliation typically significantly reduces sample area, yields nanosheets with a broad thickness distribution, and is not well aligned with commercial scalability. A bottom-up approach, where large single crystals are grown directly on technologically relevant substrates, would significantly broaden α-MoO_3_ experimental possibilities. In this pursuit, α-MoO_3_ thin films have been grown using a variety of techniques including sputter deposition,^27^ pulsed laser deposition (PLD),^28^ and atomic layer deposition (ALD) with postgrowth annealing;^29^ however, large-area single crystal morphologies have remained elusive when using these techniques. Modified PVT growth on Si substrates can produce single crystals, but typically as small (10s of μm) nanoplates or microplates which grow at inclined angles, requiring layer transfer to achieve planar sample geometries^18–20,30–32^ with few exceptions.^33^ In contrast, growth on muscovite mica and other layered substrates can promote thin, planar nanosheets.^34,35^ Notably, Molina-Mendoza Molina-Mendoza et al.^31^ synthesized singe-crystal α-MoO_3_ sheets on mica with centimeter-scale lateral sizes using a close-space sublimation method; however, tall “mesas” were distributed across the surface, precluding such crystals from use in many electronic and nanophotonic applications. A controllable growth strategy to manipulate nanosheet dimensions and morphologies is thus necessary to unlock thin, uniform, and large-area α-MoO_3_ single crystals.

By adding alkali salts to the chemical vapor deposition (CVD) growth of TMDs, the single-crystal growth rate, lateral size, and preferred texture can be enhanced through a variety of reported mechanisms.^36–40^ Of these, vapor–liquid–solid (VLS) growth, which mediates crystal growth through (in this case) liquified eutectics of alkali and transition metal compounds, is a particularly capable means to improve nanosheet morphology.^36,40^ The chemical and structural similarities between TMDs and α-MoO_3_ suggest that similar strategies may be applicable to the latter as well. Sheng et al. used alkali salts to vary α-MoO_3_ morphology from nanobelts to microtowers;^41^ due to the relatively low temperatures used, these growth modes were attributed mainly to vapor–solid–solid (VSS) mechanisms, and single-crystal morphologies were not achieved. Recently, Deng et al. used alkali compounds to grow nonlayered MoO_2_ single crystals to large sizes through the formation of a molten flux.^42,43^ These observations allude to the value in exploring salt-assisted α-MoO_3_ growth: by precisely deploying alkali salts to enable a molten intermediate phase, we may unlock a powerful VLS growth mechanism to mediate single-crystal α-MoO_3_ growth. In this work, we demonstrate ultrasmooth, high-quality α-MoO_3_ single-crystal nanosheets with lateral dimensions reaching several millimeters grown directly on A-plane α-Al_2_O_3_ (sapphire) substrates. We achieve this by using salt-assisted chemical vapor deposition (SA-CVD), which introduces a VLS growth mode capable of producing single-crystal nanosheets across a range of thicknesses. The high-quality nanosheet morphologies starkly contrast to the small, inclined microplate morphology of alkali-free α-MoO_3_ growth on sapphire. We control the growth parameters, particularly the MoO_3_ precursor flux and substrate choice, to manipulate nanosheet size and morphology and propose a self-expanding VLS-type growth model. By using scattering-type scanning near-field optical microscopy (s-SNOM), we also evaluate the launching and propagation of HPhPs in the nanosheets. Our findings introduce an exfoliation-free method for planar, large-scale, single-crystal α-MoO_3_ growth to unleash new experimental possibilities, particularly for IR nanophotonics.

## Results and Discussion

We grow α-MoO_3_ nanosheets on A-plane (112̅0) sapphire substrates using the SA-CVD method shown in Figure 1a, wherein solid-state α-MoO_3_ and NaCl are coevaporated in a horizontal tube furnace equipped with three heating zones to independently control the α-MoO_3_ source (Zone 1), NaCl source (Zone 2), and substrate (Zone 3) set point temperatures ( $T_{{\mathrm{MoO}}_{3}}$ , T_NaCl_, and T_substrate_, respectively). O_2_ and Ar flow rates are regulated using mass flow controllers (MFCs) to control the gaseous environment and assist mass transport. We show the temporal temperature profile of the three furnace zone set points over the course of a typical growth run in Figure 1b. Details of the nanosheet growth process are included in the Methods section.

**Figure 1:**
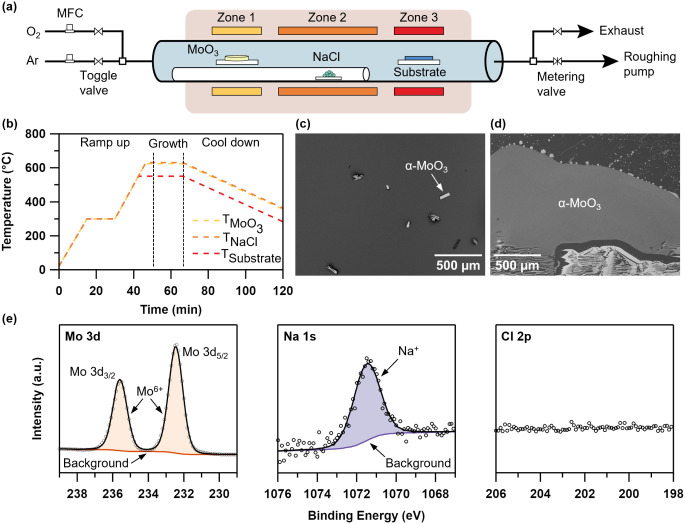
Alkali salt-assisted CVD growth of ultralarge α-MoO_3_ nanosheets. (a) Schematic of the three-zone growth chamber. (b) Programmed temperature profile of the heating zones during a typical growth run, with $T_{{\mathrm{MoO}}_{3}}$ = 625 °C, T_NaCl_ = 630 °C, and T_substrate_ = 550 °C. During the growth stage, the chamber is at 10 Torr while flowing 350 sccm O_2_ (see Methods). (c, d) SEM images of α-MoO_3_ on A-plane sapphire substrates grown (c) without and (d) with a NaCl evaporative source. (e) XPS measurements of the Mo 3d, Na 1s, and Cl 2p core level spectra taken of the α-MoO_3_ nanosheet surface.

When grown without a NaCl sublimation source, α-MoO_3_ nucleates following a 3D growth mode. This is demonstrated by the scanning electron microscopy (SEM) image in Figure 1c, where small, thick, and inclined microplates with lateral dimensions limited to ∼100 μm are observed. When the NaCl source is added under otherwise identical growth parameters, the α-MoO_3_ morphology fundamentally transforms into planar, ultrasmooth nanosheets as shown in Figure 1d, with mm-scale lateral dimensions up to 6 mm as shown in the Supporting Information (Figure S1). These nanosheets, which often incompletely cover the substrate, can vary significantly in size, thickness, and morphology across the substrate surface due to precursor concentration gradients during growth. Plan-view selected area electron diffraction (SAED) performed on a nanosheet following transfer onto a Cu TEM grid confirms the α-MoO_3_ crystal structure (Figure S2). The nanosheets are single crystalline on both the nano- to microscale, as indicated by SAED (Figure S2), and on the macro-scale, as confirmed by the electron backscatter diffraction (EBSD) mapping shown in Figure S3. No preferential in-plane orientation of the nanosheets with respect to the substrate lattice is observed, as qualitatively shown in the polarized light micrograph in Figure S4. The SA-CVD-grown crystals also often display abnormal shapes and rounded edges without preferential faceting. Whiskers, residues, and circular features resembling solidified droplets occur on the sheet edges and on the bare substrate. As we discuss below, these characteristics indicate the dominant role of a liquid phase during the growth. For the remainder of this report, all α-MoO_3_ samples are produced using the SA-CVD method unless specified otherwise.

We investigated the surface chemistry of the α-MoO_3_ nanosheets using X-ray photoelectron spectroscopy (XPS). The core level spectra of Mo 3d, Na 1s, and Cl 2p are shown in Figure 1e. The Mo 3d core level spectrum can be deconvoluted into a single doublet with peaks located at 235.6 and 232.4 eV, corresponding to the 3d_5/2_ and 3d_3/2_ spin–orbit components of Mo^6+^.^44^ We observe no additional components corresponding to lower Mo oxidation states, indicating the α-MoO_3_ crystals are approximately stoichiometric. A modest Na peak (single peak at 1071.5 eV corresponding to Na 1*s*_1/2_)^45^ is detected on the crystal surface, although the Cl signal is below the instrument detection limit. This agrees with salt-assisted growths of other layered materials and highlights the participation of Na species during the growth.^38,46^

A broad range of nanosheet thicknesses *d* can be obtained using the SA-CVD method. Figure S5 shows atomic force microscope (AFM) topographic maps of several nanosheets with *d* = 6.4–480 nm. The large-area AFM map shown in Figure S5c also highlights the uniformity of each nanosheet surface on large length scales. In Figure 2a, the Raman spectra of nanosheets with varying thicknesses are compared to a bulk α-MoO_3_ crystal grown using a PVT method, which is described in a separate report.^47^ The intensity of each spectrum was normalized to the α-MoO_3_ A_g_ (819 cm^–1^) Raman peak to account for differences in thickness. All peaks correspond to the Raman modes of either α-MoO_3_ or the sapphire substrate,^48^ with no evidence for secondary phases or Na-intercalated α-MoO_3_.^49^ The SA-CVD α-MoO_3_ line widths across all thicknesses agree with those of the bulk crystal, indicating high structural quality regardless of thickness. Due to our experimental scheme, wherein the incident laser is polarized along the α-MoO_3_ *a*-axis (see Methods), certain in-plane anisotropic α-MoO_3_ Raman modes have quenched intensities in the bulk and thick nanosheet spectra. However, as nanosheet thickness decreases, the relative intensities change and typically weak α-MoO_3_ modes emerge at 98 cm^–1^, 129 cm^–1^, 246 cm^–1^, and 295 cm^–1^.^50^ In contrast, other bands like A_g_ (82 cm^–1^), B_g_ (116 cm^–1^) and B_g_ (283 cm^–1^) see their intensities relative to A_g_ (819 cm^–1^) diminish. Similar thickness- and size-dependencies of Raman modes have previously been observed in α-MoO_3_^34^ as well as in other in-plane anisotropic vdW materials like BP,^51^ GaTe,^52^ and SnS,^53^ although understanding the responsible mechanism(s) in α-MoO_3_ is still an area of investigation^54,55^ and is beyond the scope of this work. We note that as thickness decreases, the intensity ratio of the Raman bands at ∼283 cm^–1^ and ∼295 cm^–1^ decreases. A similar trend has previously been associated with increasing oxygen vacancies.^56^ However, the fully oxidized Mo state observed in XPS of the 15 nm-thick nanosheet in Figure 1e and the enhancement of other modes in parallel with the 295 cm^–1^ Raman band suggest that the intensity ratio, which is evidently thickness-dependent, may not necessarily indicate oxygen deficiency.

**Figure 2:**
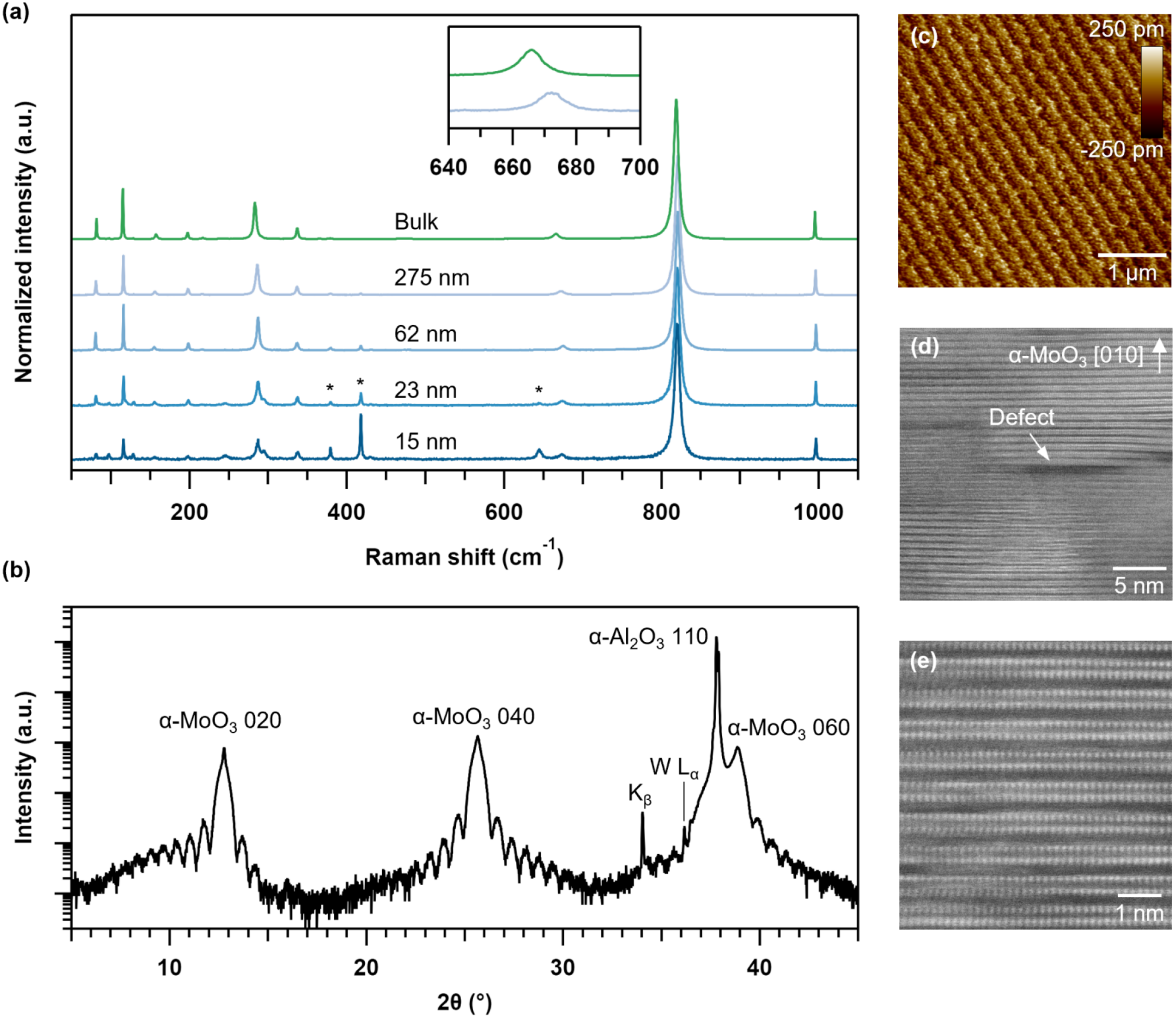
Structural characterization of the α-MoO_3_ nanosheets. (a) Normalized, offset Raman spectra of α-MoO_3_ nanosheets with varying thicknesses compared to a bulk α-MoO_3_ crystal (*d* = 2.9 μm). * = Raman peaks from the A-plane sapphire substrate. (inset) Detailed view of the B_2g_/B_3g_ (∼665 cm^–1^) band of the *d* = 275 nm nanosheet and bulk crystal. (b) Micro-XRD θ–2θ scan and (c) AFM height image of the *d* = 15 nm nanosheet. (d, e) Cross-sectional STEM HAADF images of a *d* = 100 nm nanosheet along the [102] α-MoO_3_ zone axis at two different magnifications.

Certain SA-CVD α-MoO_3_ Raman peaks exhibit an appreciable shift compared to the bulk crystal; illustratively, the B_2g_/B_3g_ (∼665 cm^–1^) band, corresponding to the asymmetric O–Mo–O stretch along the *c*-axis,^56^ is blue-shifted by about 8 cm^–1^. This could indicate compressive strain along the α-MoO_3_ *c*-axis^57^ likely induced by the coefficient of thermal expansion (CTE) mismatch with the substrate: α-MoO_3_ displays a negative CTE along the *c*-axis,^58^ significantly smaller than the CTE of sapphire of (7–8) × 10^–6^ °C^–1^.^59^ As we discuss below, this residual compressive strain can cause nanosheet buckling. In Supporting Note 2 and Figure S7, we describe a method to relieve this strain by implementing a water-based transfer technique^60,61^ that uses a spin-coated polymer support layer to transfer α-MoO_3_ nanosheets onto arbitrary substrates. The Raman spectrum of the nanosheet after transfer onto Si shows that the shifted peaks have returned to approximately their bulk values, confirming strain as the origin of the shifts. Additionally, by XPS we find that the wet transfer process effectively removes the Na signal, suggesting that, besides the macro-scale residual droplets and fronts on nanosheet edges, the Na residue is restricted to the nanosheet surface and does not penetrate the bulk α-MoO_3_ at measurable quantities. Furthermore, the transferability of the SA-CVD nanosheets without strain or alkali contamination is promising for implementing α-MoO_3_ single crystals in a wide range of structures and devices.

We collected an X-ray microdiffraction (micro-XRD) θ–2θ scan on a single α-MoO_3_ nanosheet, which agrees with the expected *b*-axis texturing of the α-MoO_3_ crystal structure (Figure 2b). The appearance of Pendellösung fringes indicate high crystal quality and smooth interfaces. The AFM height image of the same nanosheet (Figure 2c) exhibits a clean step-and-terrace morphology that matches the underlying substrate, indicating high-quality step-flow growth. The surface morphology is sensitive to the growth parameters: NaCl-rich conditions can result in particulates overtop the nanosheet surface, as shown in Figure S5a.

Cross-sectional scanning transmission electron microscopy (STEM) of a nanosheet with *d* = 100 nm reveals well-ordered vdW layers with an interplanar spacing of ∼0.7 nm, corresponding to 1/2 of the α-MoO_3_ *b*-axis lattice parameter (Figure 2d,e). Because of the random in-plane orientation of the nanosheets, the high-symmetry α-MoO_3_ axes did not align with those of the substrate and required sample realignment to resolve atomic columns. Throughout the imaged region, we observe scattered linear horizontal defects up to ∼25 nm in length that may represent edge dislocations and/or layer twisting or splitting (Figure 2d). Energy-dispersive X-ray spectroscopy (EDS) maps of the entire nanosheet cross-section are shown in Figure 3a and indicate uniform Mo and O concentrations throughout. We note that the relatively high noise level in the Na map is due to overlap of the Na Kα EDS peak with Cu Lß and Ga Lα, which are common contaminants from the TEM grid and lamella preparation, respectively. Interestingly, a thin Na-rich layer is present at the sapphire/α-MoO_3_ interface, emphasized in the extracted EDS spectra in Figure S8 and confirmed by XPS depth profiling in Figure S9. This Na-rich interface rapidly undergoes damage when imaged at high magnifications: as shown in Figures S10 and S11, the large electron dose rate induces highly ordered crystal growth of a new interfacial phase that rapidly grows in volume and distorts the nearby α-MoO_3_ layers. This electron-beam-induced crystal growth presently hampers our ability to study the pristine bottom interface using high-resolution STEM and is currently under further investigation. We also note that the entire α-MoO_3_ nanosheet was susceptible to beam damage during STEM sample preparation, causing variations in the STEM contrast that are visible in Figure 3a and discussed in Supporting Note 3 and Figure S12.

**Figure 3:**
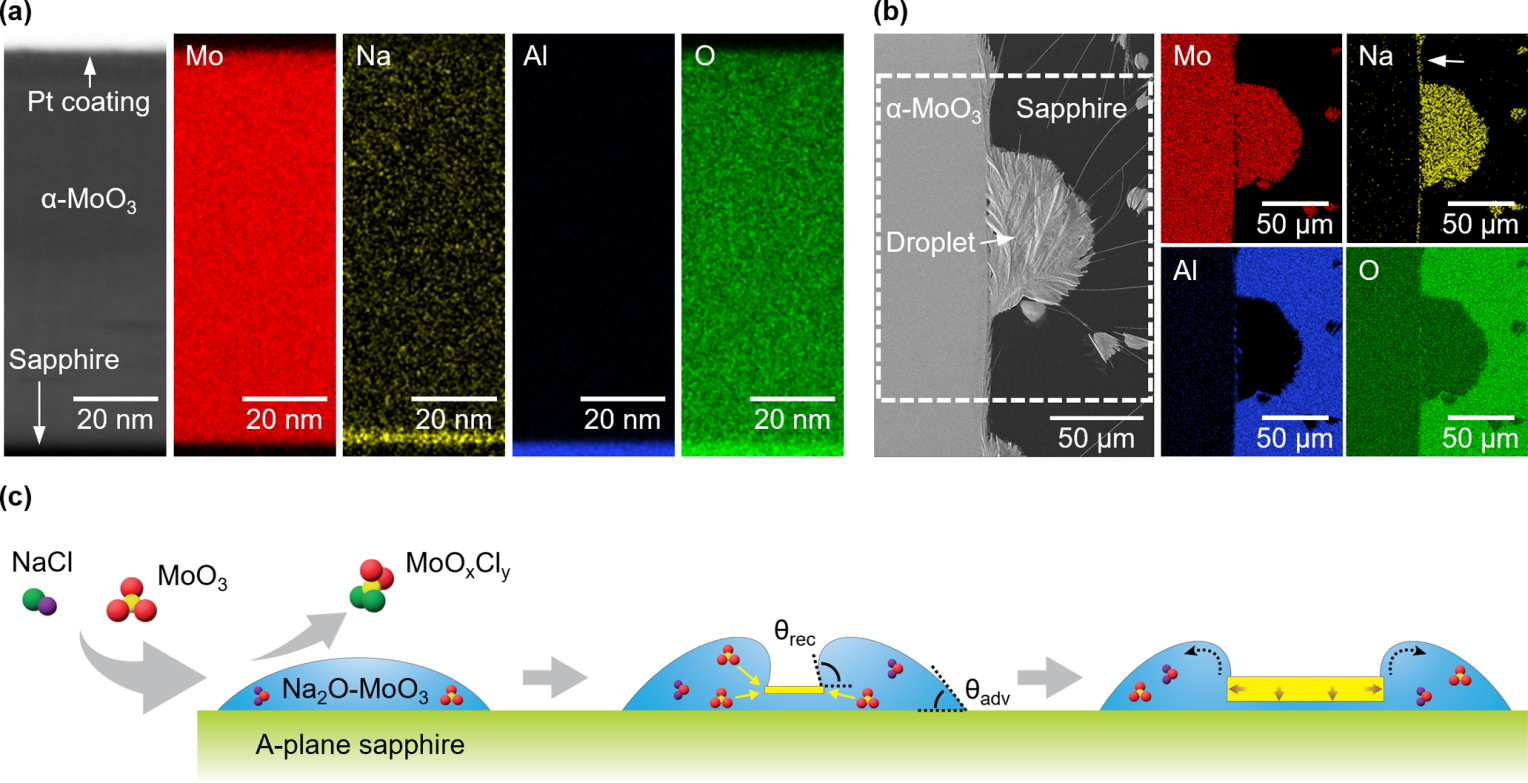
Self-expanding VLS growth mode. (a) Cross-sectional STEM HAADF image and EDS maps of the α-MoO_3_ nanosheet. (b) SEM image and EDS maps taken from the outlined region of a nanosheet edge and solidified droplet. The arrow points to a Na-rich front along the nanosheet margin. (c) Schematic of the proposed laterally self-expanding VLS growth mode.

To explore the growth mechanism(s) responsible for the improved morphologies obtained from SA-CVD growth, we inspect the nanosheets using SEM-EDS. The solidified residues of a liquid phase, including droplets and whiskers, are often observed on the samples that exhibit large, smooth nanosheets as shown in Figure 3b. EDS analysis reveals that these residues belong to the Na_2_O–MoO_3_ system, often with Na/(Na + Mo) = 0.33–0.38 (Figure S13 and Table S1). This composition is slightly Na-rich compared to the low-melting-temperature eutectic composition in the Na_2_MoO_4_–MoO_3_ phase diagram.^62^ Presumably, the excess Na content results from the continual depletion of MoO_3_ from the molten intermediate during growth. The Na_2_O–MoO_3_ residue is also observed as a “front” along some margins of the nanosheets, as shown by the thin line of Na along the nanosheet edge in Figure 3b and may mediate lateral growth.

We propose a growth model similar to the one introduced by Jiang et al.^40^ which combines dual effects in the case of NaCl-assisted WSe_2_ monolayer growth: (I) a molten intermediate interlayer separating the substrate and the growing nanosheet, and (II) a VLS “crawling” mechanism that mediates precursor incorporation into the nanosheet edges through a self-expanding molten front. Based on our observations and illustrated in Figure 3c, NaCl and MoO_3_ first react on the substrate surface to produce a liquid Na_2_O–MoO_3_ intermediate, as the substrate temperature (∼590 °C, Figure S14) exceeds the eutectic melting point of 540 °C.^62^ As no Cl is detected in the as-grown samples, we believe it exits the growth chamber as volatile MoO_*x*_Cl_*y*_ compounds, as has been proposed for NaCl-assisted TMD growth.^37,38,63^ As additional precursor vapors are incorporated into the melt, supersaturated MoO_3_ nucleates at the melt top surface. The liquid front then travels laterally, incorporating additional MoO_3_ into the nanosheet to enable lateral growth. This self-expanding operation is driven by interfacial free energy differences: the vdW bonding of the α-MoO_3_ causes a small droplet/α-MoO_3_ interfacial free energy compared to that of the droplet/sapphire interface, producing a larger contact angle between the receding droplet/α-MoO_3_ interface (θ_rec_) than the advancing droplet/sapphire interface (θ_adv_). As a result, an unbalanced Young’s force^64^ accelerates droplets laterally as the nanosheets grow, resulting in self-expanding continuous growth.^36^ In contrast to most WSe_2_ nanosheets in the aforementioned Jiang et al. study,^40^ the α-MoO_3_ nanosheets in this work are multilayered and can reach hundreds of nanometers thick. A self-limiting mechanism, likely arising from the requirement for reducing agents like H_2_, typically promotes monolayer growth in the WSe_2_ case;^40^ however, this mechanism can be overridden by laterally confining the melt during growth, increasing melt-borne H_2_ and Se concentrations and producing thick WSe_2_ nanoplates.^65^ In our α-MoO_3_ growth, the multilayered growth may result from the absence of an analogous self-limiting mechanism; indeed, Mo retains its maximum valence state (6+) in MoO_3_, allowing continuous vertical growth without reducing agents. The ultrasmooth and uniform α-MoO_3_ topography regardless of thickness suggests that, like lateral expansion, ideal vertical growth also occurs through the VLS mechanism. Therefore, a mechanism where the nanosheet grows from the top down as melt-borne precursors attach from underneath is most compatible with our observations. On cooling, the remaining melt solidifies at the nanosheet/substrate interface and as frozen droplets, fronts, and whiskers on the nanosheet edges and substrate. We find that these residues are not readily removed by solvent rinsing or thermal annealing—which furthermore may damage the α-MoO_3_—and may impact device fabrication and performance if not eliminated by techniques like wet layer transfer.

The SA-CVD growth is highly sensitive to both the MoO_3_ and NaCl precursor flux, although we find that under our experimental conditions, the former more strongly controls nanosheet morphology. SEM images in Figure 4 show the morphology evolution of samples grown using $T_{{\mathrm{MoO}}_{3}}$ = 630 °C, 640 °C, 645 °C, and 650 °C with constant T_NaCl_ = 650 °C and T_substrate_ = 570 °C. The α-MoO_3_ nanosheets form narrow dendrites at $T_{{\mathrm{MoO}}_{3}}$ = 630 °C and more compact morphologies at $T_{{\mathrm{MoO}}_{3}}$ = 640 °C. At $T_{{\mathrm{MoO}}_{3}}$ = 645 °C, however, several disparate morphologies coexist on the substrate: (I) thin dendrites like those present at 630 °C; (II) large, ultrasmooth nanosheets of varying thicknesses spanning hundreds of micrometers; and (III) thick nanosheets covered with tall, faceted mesas (Figure S15). The mesas introduce significant roughness to the α-MoO_3_ surface, which may scatter HPhPs and degrade performance. At $T_{{\mathrm{MoO}}_{3}}$ = 650 °C, the α-MoO_3_ crystals completely cover the substrate and overwhelmingly exhibit the mesa-dominated morphology. The mesas can exceed 1 μm in height and establish a significant increase in thickness; these thick crystals often buckle after growth on cooling to room temperature. Buckling generally occurs perpendicular to the α-MoO_3_ *c*-axis as identified by the crystal habit: the facets along the α-MoO_3_ *c*-axis are longer than those along the *a*-axis due to growth rate anisotropy in non-VLS growth modes.^25,34^ This buckling reinforces our assessment of CTE-mismatch-induced compressive stress along the *c*-axis of α-MoO_3_. Buckling may also occur after weeks or months of exposure to humid environments. We believe that over time, water molecules infiltrate the nanosheet–substrate interface and weaken the adhesion or dissolve the Na-rich layer, eventually causing destructive strain relaxation. Although buckling could impede certain properties and interrupt HPhP propagation, it is rarely observed in thin nanosheets and can be largely circumvented in all but extremely thick nanosheets by relieving internal strains through postgrowth layer transfer (Figure S7).

**Figure 4:**
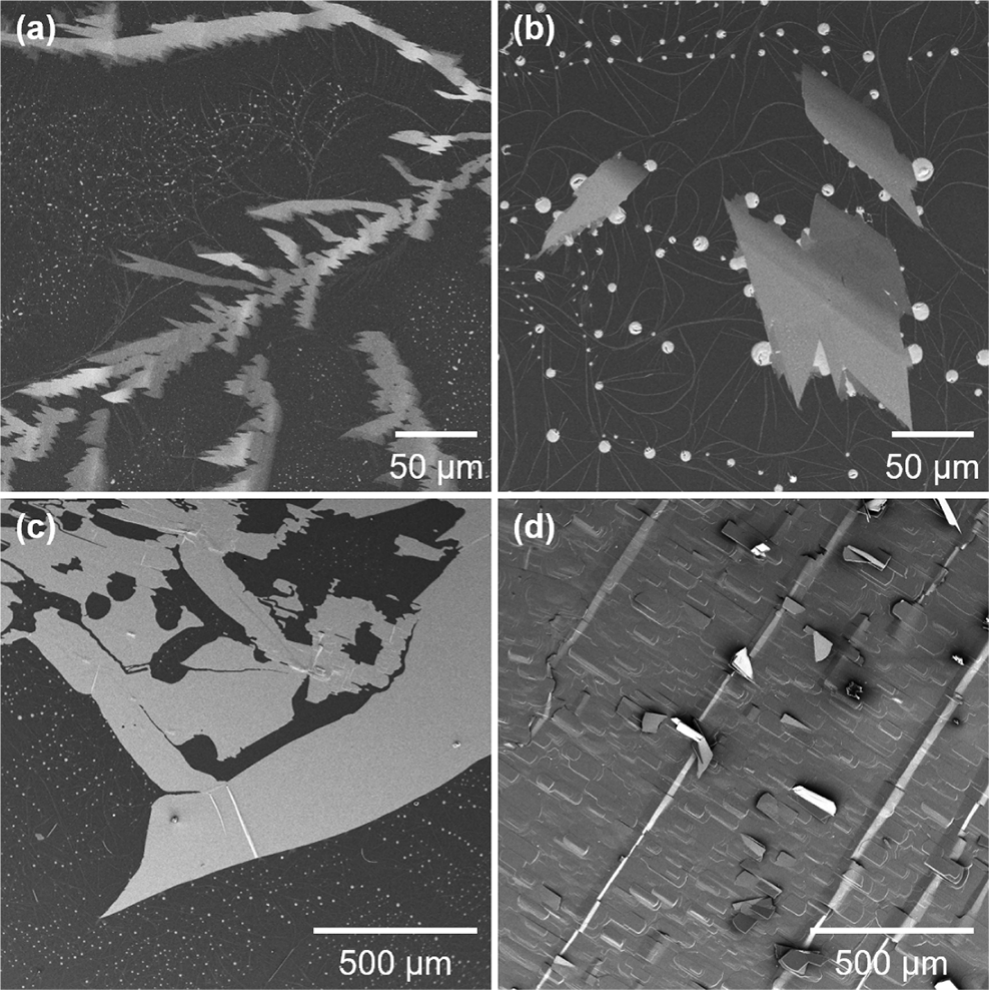
Nanosheet morphology dependence on precursor MoO_3_ flux. SEM images of α-MoO_3_ nanosheets grown using a $T_{{\mathrm{MoO}}_{3}}$ set point of (a) 630 °C, (b) 640 °C, (c) 645 °C, and (d) 650 °C.

Similar morphology trends can be provoked by varying the mass of the α-MoO_3_ source, which is an alternative pathway to modify the sublimation rate (Figure S16). We also verified the high growth sensitivity to the MoO_3_ precursor flux by repeating nominally identical growths, which indicate that the baseline variability of the deposition process is relatively small compared to the significant morphological differences evoked by changing the MoO_3_ flux (Figure S17). These morphological evolutions can be understood as deviations from the ideal self-expanding VLS mode. The dendritic structures that form at low MoO_3_ vapor flux (Figure 4a,b) are consistent with mass-flow-limited growth modes reported in other systems.^66^ By growing in this regime, we can achieve ultrathin layers with *d* ≈ 6 nm (Figure S5a). However, as shown in Figures S15 and S16, α-MoO_3_ rapidly decomposes under the growth conditions, preventing these low-growth-rate nanosheets from reaching large sizes regardless of growth duration. At moderate MoO_3_ flux (Figure 4c), the VLS growth rate exceeds the decomposition rate to enable large-area nanosheets with moderate thicknesses. When the MoO_3_ flux becomes too large (Figure 4d), high MoO_3_ concentrations in the melt rapidly increase nanosheet thickness. Additionally, the appearance of faceted mesas, while VLS-grown crystals are facet-free, suggests the emergence of a secondary growth mode. Mesas are often observed in α-MoO_3_ grown by vapor deposition without intentional alkali additives,^31,35^ suggesting that in our case, they likely nucleate either directly from the vapor (vapor–solid growth; VS) or assisted by unmelted alkali-containing surface compounds (vapor–solid–solid growth; VSS). The coexistence of multiple morphologies on a single sample likely results from spatial and temporal variations in precursor fluxes, common in solid-source vapor transport techniques;^67^ in future experiments, gas-phase precursor control may be useful to more precisely regulate nanosheet growth.

We performed simultaneous SA-CVD growth on several different substrates (A- and C-plane sapphire, Si (100), and muscovite mica). The substrate strongly controls the resulting α-MoO_3_ morphology, where we observe smooth, large-area nanosheets only on A-plane sapphire substrates. More information is available in Supporting Note 7 and Figure S20. Since the droplet/substrate interfacial free energy drives the self-expanding VLS growth mode, we believe that the high A-plane sapphire surface energy^68,69^ facilitates high-quality growth. Large-area but mesa-covered α-MoO_3_ nanosheets are obtained on mica substrates regardless of whether NaCl is added, suggesting that the intrinsic alkali content (both K and Na, Figure S21a,b) in commercial mica substrates might be integral to large-area α-MoO_3_ growth on mica.^31,34^ This conclusion is supported by the surfactant-like “floating” behavior of mica-derived Na during α-MoO_3_ growth (Figure S21c) and by recent work showing similarly alkali-assisted growth of MoO_2_ on mica.^42^ We also explored an alternative, two-step alkali salt-assisted growth process, detailed in Supporting Note 8 and Figure S22. Here, we spin coated NaOH onto the substrate as the alkali precursor before vapor growth of α-MoO_3_ with no NaCl sublimation source. However, this technique failed to produce single-crystal samples even after an extensive growth campaign, instead favoring noncrystallographically branched morphologies.

Finally, we performed near-field optical measurements to assess the suitability of the α-MoO_3_ nanosheets for nanophotonic applications. Using s-SNOM, polaritons can be excited with an IR beam of a desired frequency through elastic scattering from a metallized AFM tip or crystal edge,^70,71^ where the latter is dominant in our setup. More information on the near-field measurements is available in Methods. For efficient HPhP launching, straight and low-index crystal edges are preferred; although such faceting rarely occurs in the SA-CVD nanosheets, Figure 5a shows an optical micrograph of one such nanosheet (*d* = 230 nm) with a straight edge misaligned by only ∼7.5° from α-MoO_3_ [001]. We note that the misalignment, evaluated in Figure S23a, may modestly affect the HPhP excitation efficiency and propagation characteristics in accordance with previous reports.^72,73^ Nevertheless, we find that the edge effectively excites fundamental-mode HPhPs under s-SNOM interrogation within the second α-MoO_3_ Reststrahlen band, where in-plane anisotropic polaritons propagate in the [100] direction.^9^ This is evident by the bright, frequency-dependent HPhP interference fringes near the edge in the near-field amplitude (S_3_) images shown in Figure 5b. The excitation frequencies are swept from 915 cm^–1^ until the HPhPs no longer propagate at 960 cm^–1^, with the remaining frequency maps shown in Figure S24a. In addition to edge-launched polaritons, isolated point-like launching sites are sometimes observed in the near-field amplitude images (Figure 5c), but do not appear in topography scans as shown in Figure S23b. We suspect that subsurface defects—potentially the horizontal defects observed in the atomic resolution STEM images of Figure 2d—may constitute these scattering sites. Although outside the scope of this work, further studies are underway to characterize the defect-launched HPhPs and assess their impact on polariton propagation.

**Figure 5:**
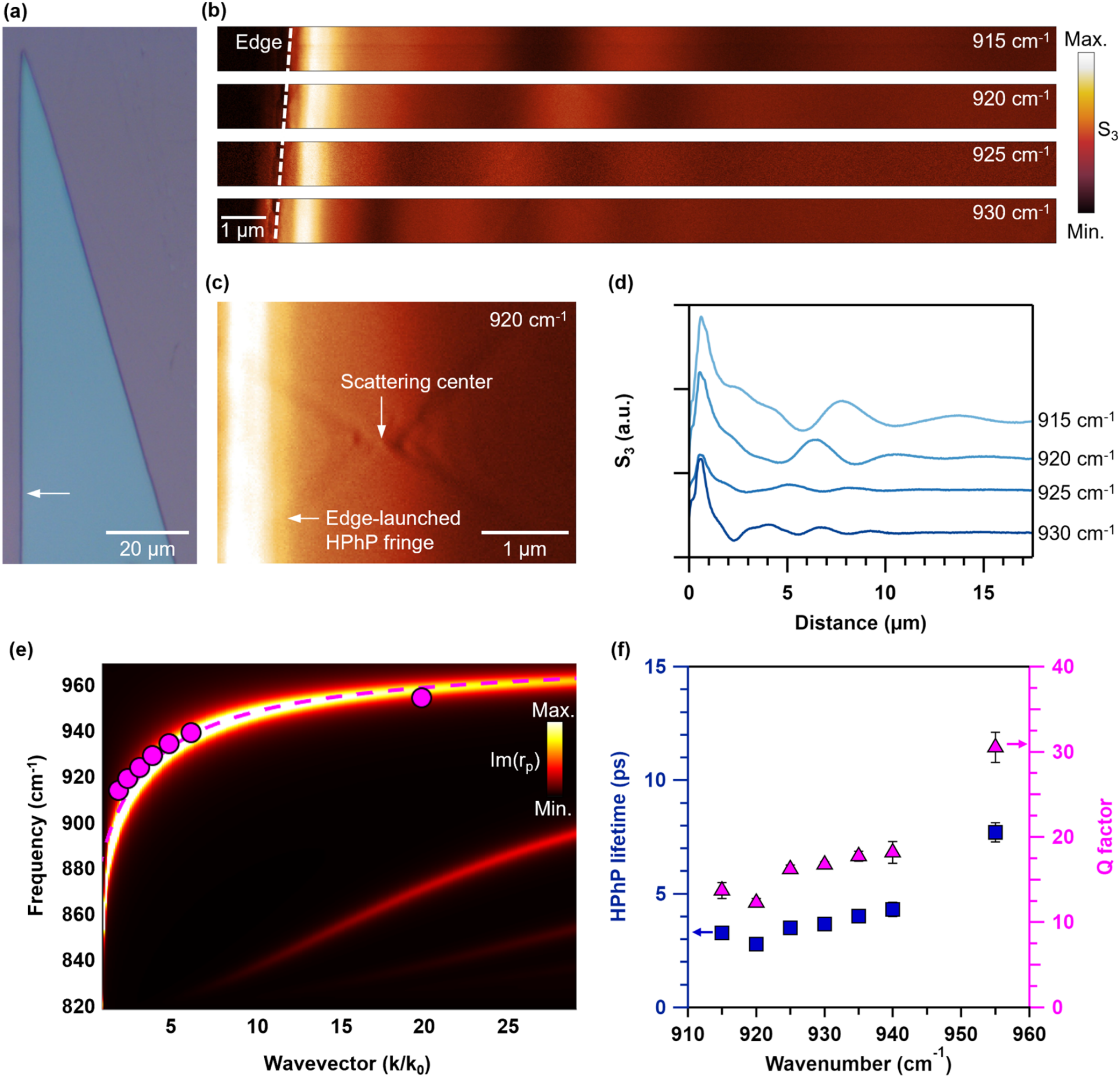
Hyperbolic phonon polariton modes of SA-CVD α-MoO_3_ on A-plane sapphire. (a) Optical micrograph of the 230 nm-thick nanosheet probed by s-SNOM. (b) s-SNOM near-field amplitude (S_3_) images taken at different IR frequencies between 915 and 930 cm^–1^ along the edge marked by the arrow in (a). (c) s-SNOM image of a nontopographic feature launching HPhP modes. (d) Line profiles extracted from the amplitude fringes in (b). (e) TMM-calculated (false color map) and experimental (markers) dispersion relations of the HPhP modes along the α-MoO_3_ [100] direction. (f) Lifetimes (squares) and Q factors (triangles) of the HPhP modes.

Horizontal line profiles of the near-field amplitude maps from 915 to 930 cm^–1^ are shown in Figure 5d, with additional frequencies up to 955 cm^–1^ reported in Figure S24b. We extract the complex HPhP wavevector (*k*) from the s-SNOM maps by taking the fast Fourier transform (FFT) of the line profiles (see Methods). The experimental dispersion points, ω­(Re­[*k*]/*k*_0_), are overlaid in Figure 5e on the analytical biaxial dispersion (dashed line) and transfer-matrix method (TMM)-calculated Im­(r_p_) (contour plot) using the reported dielectric function of α-MoO_3_.^74^ Our experimental and theoretical data agree, confirming that the SA-CVD nanosheets preserve the mid-IR optical properties of α-MoO_3_ grown by other techniques. Compared to nondispersive substrates like Si, the near-zero permittivity of the sapphire substrate in this frequency range^75^ significantly modifies the HPhP dispersion by shifting the fundamental HPhP branch closer to the light line (ω­(Re­[*k*]/*k*_0_) = 1). Hence, our investigations on the HPhP modes supported in α-MoO_3_ nanosheets are limited to the frequency range where the long-wavelength polaritons do not support an edge-to-edge Fabry–Perot cavity^76,77^ and where sapphire has a positive real permittivity, as necessary to prevent polariton hybridization with the surface phonon polaritons supported in the substrate.^75,78^ From the Im­(*k*) values fitted from our measurements, we calculated the HPhP propagation lengths $\left(L_{p}=\frac{1}{Im\left[k\right]}\right)$ shown in Figure S24c. The FFT-extracted lifetimes $\left(\tau =\frac{L_{p}}{v_{g}}\right)$ , derived from the HPhP group velocity $\left(v_{g}=\frac{\partial \omega }{\partial Re\left[k\right]}\right)$ of the analytical dispersion, and quality factors (Q=2πRe[k]Im[k]) of the edge-launched polaritons are shown in Figure 5f. The lifetimes increase from 2.8 to 7.7 ps and the Q factors from 12 to 30 throughout the measured frequency range. The phonon polariton resonances and losses arising from the sapphire substrate make a direct comparison of intrinsic material quality to literature reports imprecise. Regardless, we report lifetimes and Q factors that are of sufficient magnitude for various nanoscale investigations and are comparable with suspended^79^ and SiO_2_/Si-supported^9,80^ α-MoO_3_ crystals produced by other methods. Therefore, the SA-CVD method is well suited for synthesizing large-area α-MoO_3_ nanosheets for HPhP-based nanophotonics while circumventing exfoliation processes.

## Conclusion

This work introduces an SA-CVD method on A-plane sapphire substrates to fabricate large-area, ultrasmooth, single-crystal α-MoO_3_ nanosheets directly on a substrate for the first time, bypassing exfoliation steps. This favorable morphology is enabled by adding a NaCl evaporative source to an α-MoO_3_ PVT process and tightly controlling the precursor vapor concentrations via multizone temperature control. Then, a self-expanding VLS-type growth mode mediates the lateral and vertical growth of uniform α-MoO_3_ nanosheets. The nanosheets are single crystalline, can exceed several millimeters in lateral area, and enjoy high structural quality. The α-MoO_3_ morphology is particularly sensitive to the precursor MoO_3_ vapor concentration and the choice of substrate. We excite low-loss HPhPs in the nanosheets to demonstrate the suitability of this growth technique for large-area nanophotonics studies. Therefore, despite the presence of alkali-containing residue at the nanosheet interfaces—which can furthermore be removed by wet layer transfer—the SA-CVD method produces high-quality single crystals without degrading HPhP propagation. These results unlock new possibilities and perspectives for synthesizing large-area, single-crystal α-MoO_3_—and potentially other vdW transition metal oxides that currently lack practical bottom-up synthesis techniques—through alkali salt growth additives.

## Methods

### SA-CVD Growth of α-MoO_3_ Nanosheets

A three-zone horizontal tube furnace (Thermcraft XST-3-0-24-3 V2-F02) equipped with a 50 mm-outer-diameter (OD) fused silica (SiO_2_) tube, shown in Figure 1a, is used to grow the α-MoO_3_ nanosheets. Several process refinements were adopted to manage challenges with process inconsistency, which likely arise from variable vapor fluxes from the solid-state sources and from unintentional sublimation and redeposition of deposits from the tube walls onto the substrate. Rather than using a powder for the MoO_3_ source, we used pressed α-MoO_3_ pellets to minimize sublimation rate variability caused by surface area shrinkage during growth. We also implemented a tube-inside-a-tube geometry, placing the NaCl source within a secondary 14 mm-OD SiO_2_ tube, to prevent cross-contamination of the sources and promote steady sublimation rates.^81^ Prior to each deposition, the SiO_2_ tubes were baked out at 900 °C for 20 min and the substrate mounting plate grit blasted to remove volatile deposition products.

For the α-MoO_3_ sublimation source, approximately 4 g of α-MoO_3_ powder (Alfa Aesar, 99.9%) is pressed into a 25.4 mm-diameter pellet, sintered in air at 700 °C for 2 h, and placed on a fused SiO_2_ plate in the center of Zone 1 of the furnace. The pellet surface area, and thus sublimation rate, changes only modestly throughout each deposition, allowing its usage for multiple growth runs. Twenty mg of NaCl powder (Sigma-Aldrich, 99.999%) is placed in a Pt boat in Zone 2 within the inner tube for protection from cross-contamination. A- and C-plane sapphire (MSE Supplies) and Si (100) substrates are prepared by spin washing with deionized water, isopropyl alcohol, and methanol followed by ultraviolet ozone treatment for 20 min. Muscovite mica substrates (Ted Pella, grade V1) are cleaved using a razor blade to produce a clean surface immediately before growth. The prepared substrates are placed on a SiO_2_ plate and positioned in Zone 3. The three zones are heated at 20 °C/min to 300 °C and held for 15 min at 1 atm under 50 sccm Ar flow to remove water from the reactor. In the same atmosphere, the zones are then heated at 20 °C/min to the growth temperatures; typical furnace set points for high-quality growth are $T_{{\mathrm{MoO}}_{3}}$ (Zone 1) = 625 °C, T_NaCl_ (Zone 2) = 630 °C, and T_substrate_ (Zone 3) = 550 °C. Continual adjustment of optimal temperatures is required due to the gradually changing state of the α-MoO_3_ source and the tube walls. We note that the temperature measured by an external thermocouple at the radial tube center of each zone is higher than the set point, as shown in the lateral temperature profile in Figure S14. Because of the numerous thermal gradients inherent within the furnace, for consistency we report only the set point temperatures. After reaching the set points, we stabilize the zone temperatures for ∼5 min before beginning growth. To grow the α-MoO_3_ nanosheets, we flow 350 sccm of O_2_ and evacuate the chamber to 10 mTorr to facilitate the evaporation and transport of the MoO_3_ and NaCl sources. After 15 min, we cease the gas flow, vent the chamber to 1 atm, and allow the furnace to cool to at least 300 °C before removing the samples.

### Imaging

A TESCAN MIRA instrument is used to collect SEM images and associated EDS measurements. First, ∼3 nm of W is sputter deposited on the sample surfaces to reduce charging during SEM imaging. Most SEM images were taken using a secondary electron detector with a 3 keV accelerating voltage, 100 pA beam current, and a working distance of approximately 4.5 mm. The images in Figure 1c,d were taken using a backscattered electron detector with 5 keV accelerating voltage, 100 pA beam current, and 9 mm working distance. EDS maps are collected using 6 keV accelerating voltage, 600 pA beam current, and 15 nm working distance. We use an Asylum MFP-3D AFM in tapping mode with a scan rate of 0.3–0.5 Hz to measure surface topography and nanosheet thickness. Optical micrographs were primarily collected with an Olympus BX60M microscope and polarized light micrographs using a Zeiss Axiovert 200M instrument. In some images, the brightness and contrast were adjusted using Adobe Photoshop.

EBSD studies were carried out using a Thermo Fisher Helios 5 Hydra SEM equipped with an EDAX Velocity Ultra EBSD detector. The sample was coated with ∼5 nm of conductive carbon to minimize charge accumulation. The operating voltage used was 10 kV and the current was 26 nA. The IPF maps were generated using EDAX OIM analysis software.

Samples were prepared for STEM analysis utilizing a Thermo Fisher Scientific Helios 5 UC focused ion beam (FIB) equipped with a Ga^+^ liquid metal ion source. The sample was first sputter-coated with a thin film of Pt to reduce charging during FIB prep. A region of an α-MoO_3_ flake was covered by a layer of Pt electron-beam-induced deposition followed by a thicker layer of Pt ion-beam-induced deposition. The protected region was isolated from its surroundings using high current milling at 30 kV and was then extracted using a micromanipulator and attached to a copper TEM grid using Pt ion beam-induced deposition. The resultant lamella was thinned with progressively lowering ion beam currents and voltages until it was electron transparent.

STEM analysis was performed on an image- and probe-corrected Thermo Fisher Scientific Spectra Ultra equipped with an X-FEG/UltiMono source operating at 300 keV accelerating voltage. Images were acquired using a 28 mrad semi-convergence angle on a high-angle annular dark field detector with collection range of 49–200 mrad using ∼50–90 pA beam current. EDS was acquired on a six-segmented windowless UltraX detector with a 4.45 srad solid angle using ∼150 pA beam current. STEM-EDS spectral maps were processed within Velox software by applying a 4-pixel average prefilter to the data set to reduce the influence of noise prior to generating weight percent maps. In some images, gamma correction was performed using Adobe Photoshop to improve visibility.

### X-ray Diffraction

XRD θ–2θ scans were performed using a Panalytical Empyrean 3 diffractometer with a Cu Kα source, Bragg–Brentano^HD^ incident optics and a PIXcel^3D^ detector with programmable antiscatter slit. For micro-XRD, we inserted a microbeam collimator with a 0.3 mm diameter opening to interrogate single α-MoO_3_ nanosheets and minimize interference from other crystals and residual droplets on the sample surface.

### Raman Spectroscopy

Raman spectroscopy is performed using a Horiba LabRAM HR Evolution Raman microscope with a linearly polarized 488 nm excitation laser with a maximum laser power of 2.4 mW focused through a 100× objective lens. The theoretical laser spot size is 0.66 μm and the spectral resolution is ∼0.8 cm^–1^ using an 1800 gr/mm grating. Because of the in-plane anisotropy of the α-MoO_3_ sheets, we position a half-wave plate between the laser and the microscope to rotate the beam polarization until the α-MoO_3_ A_g_ peak near 819 cm^–1^ was maximized. This ensures that the relative intensities of the Raman peaks remain consistent between measurements.^82^ The intensities of the Raman spectra are normalized to the A_g_ peak at 819 cm^–1^ to account for differences in thickness.

### X-ray Photoelectron Spectroscopy

XPS measurements are collected using a Physical Electronics VersaProbe II instrument and a monochromatic Al K_α_ X-ray source. Measurements are performed using a takeoff angle of 45° with respect to the sample surface, and the analysis size is ∼200 μm in diameter. The samples are charge compensated using low-energy electrons and Ar ions. Depth profiles were performed using 20 s intervals of Ar ion beam milling at 1.5 kV. The spectra were charge calibrated to the C 1s peak at 284.7 eV and fittings performed using the CasaXPS software package.

### Near-Field Characterization and Analysis

We accomplish the near-field characterization of HPhPs in the α-MoO_3_ nanosheets through subdiffractional-resolution imaging via s-SNOM.^71^ When light is scattered from either the metalized AFM tip (∼20 nm) or flake edge, HPhPs are excited from the tip and edge reflected or propagate from the crystal edge, respectively, and are scattered back to the detector.^83^ With the flake edge orthogonal to the incident light, we promote efficient edge scattering resulting in prominent edge-launched HPhPs. In our s-SNOM setup from neaspec (Attocube GmbH), the sample is excited with a tunable quantum cascade laser (QCL; MIRCAT by Daylight Solutions) which sweeps across desired frequencies in the second α-MoO_3_ Reststrahlen band and a mercury cadmium telluride (MCT) detector collects the near-field signal.

The HPhP complex wavevector is described by the biaxial analytical dispersion relation^74^

1 $$\frac{k}{k_{0}}=\frac{\rho }{k_{0}d}\left[\tan^{-1}\left(\frac{\rho \varepsilon_{a}}{\varepsilon_{z}}\right)+\tan^{-1}\left(\frac{\rho \varepsilon_{s}}{\varepsilon_{z}}\right)+\pi l\right],\;l=0,1,2...$$

where *k* is the complex in-plane HPhP wavevector, $k_{0}=\frac{\omega }{c}$ is the free space wavevector, *d* is the flake thickness, *l* is the discrete HPhP mode order (*l* = 0 corresponds to the fundamental mode), ε_a_ is the permittivity of air, ε_s_ is the permittivity of the substrate, and ε_[010]_ is the out-of-plane permittivity of α-MoO_3_. The factor $\rho =i\sqrt{\frac{\varepsilon_{[010]}}{\varepsilon_{[100]}\cos^{2}\left(\alpha \right)+\varepsilon_{[001]}\sin^{2}\left(\alpha \right)}}$ addresses the in-plane permittivities (ε_[100]_, ε_[001]_) of α-MoO_3_ and α is the sample orientation angle between the [100] direction and the in-plane incident excitation source wavevector. We extract the complex wavevector by sending the real-space line profiles from s-SNOM maps through a fast Fourier transform (FFT). The resulting FFT spectra is constituted of peaks in wavevector space depending on the frequencies present in the line profile. By fitting the Fourier peaks with a Lorentzian line shape, the peak position corresponds to the Re­[*k*] and the full width at half-maximum (fwhm) represents the approximate Im­[*k*]. We report the dispersion of the α-MoO_3_ nanosheets from the analytical dispersion (eq 1) and the Im­(r_p_) values calculated with the transfer-matrix method (TMM).^84^ We perform the TMM calculations for an air/α-MoO_3_/α-Al_2_O_3_ stack with a flake thickness of 230 nm matching the sample geometry under near-field characterization.

## Supplementary Material



## Notes

Additional details on the α-MoO_3_ nanosheet morphology; imaging by optical microscopy, polarized light microscopy, AFM, and SEM; structural and compositional characterization; EBSD, TEM, STEM, EDS, XPS, and Raman spectroscopy; characterization of SA-CVD α-MoO_3_ grown on various substrates; s-SNOM characterization of HPhP propagation at higher frequencies; experimental details for polymer-assisted wet layer transfer and two-step SA-CVD growth (PDF)
