# Supplementary material for: Millimeter-Scale Single-Crystal α‑MoO3 Nanosheets Grown by Alkali Salt-Assisted Chemical Vapor Deposition
Source: ACS Nano. 2026 Jul 9;20(28):20379–91. doi: 10.1021/acsnano.6c07396 (PMC13394530; doi:10.1021/acsnano.6c07396)
Supplement: Supplementary file 1 [file nn6c07396_si_001.pdf]

Supporting Information

For "Millimeter-Scale Single-Crystal α-MoO_3 Nanosheets Grown by Alkali Salt-Assisted Chemical Vapor Deposition"

Ryan W. Spangler,[1] Thiago S. Arnaud,[2] Caleb Whittier,[3] Bhaveshkumar Kamaliya,[3] Sai S. Tripathy,[4] Patrick E. Hopkins,[5-7] Elizabeth C. Dickey,[4] Joshua D. Caldwell,[2,8] Nabil D. Bassim,[3,9] Jon-Paul Maria*[1]*

1. Department of Materials Science and Engineering, The Pennsylvania State University, University Park, PA 16802, USA
2. Interdisciplinary Materials Science Program, Vanderbilt University, Nashville, TN 37240, USA
3. Department of Materials Science and Engineering, McMaster University, Hamilton, ON L8S 4L7, Canada
4. Department of Materials Science and Engineering, Carnegie Mellon University, Pittsburgh, PA, 15213 USA
5. Department of Mechanical and Aerospace Engineering, University of Virginia, Charlottesville, VA 22904, USA
6. Department of Materials Science and Engineering, University of Virginia, Charlottesville, VA 22904, USA
7. Department of Physics, University of Virginia, Charlottesville, VA 22904, USA
8. Department of Mechanical Engineering, Vanderbilt University, Nashville, TN 37235, USA
9. Canadian Centre for Electron Microscopy, Hamilton, ON L8S 4M1, Canada

Table of Contents

Supporting Note 1. SA-CVD α-MoO_3 morphology and structure 2
Supporting Note 2. Methods for wet transfer of SA-CVD nanosheets 7
Supporting Note 3. Additional interface characterization and STEM imaging 9
Supporting Note 4. SEM/EDS of Na_2O–MoO_3 residual droplet 13
Supporting Note 5. Temperature profile 14
Supporting Note 6. SA-CVD α-MoO_3 morphology evolution 15
Supporting Note 7. α-MoO_3 growth on different substrates 18
Supporting Note 8. Two-step SA-CVD growth of α-MoO_3 on A-plane sapphire 21
Supporting Note 9. Additional s-SNOM results 23
References 24

Supporting Note 1. SA-CVD α-MoO_3 morphology and structure

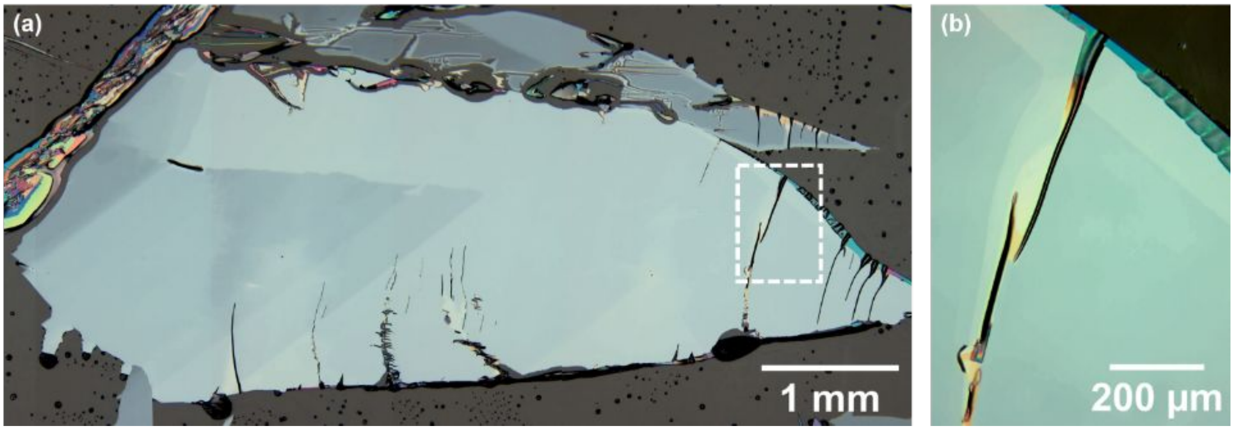

Figure S1. Large-area α-MoO_3 nanosheet grown by SA-CVD. **(a)** Stitched optical micrograph depicting an ultra-large (~2 mm × 6.5 mm) single crystal, although marked by some nonuniformities and buckling. **(b)** Close-up optical micrograph of the buckled region outlined by the dashed rectangle in (a).

We performed plan-view TEM imaging and selected area electron diffraction (SAED) to confirm the crystal structure of the SA-CVD nanosheets and that they are single-crystalline on short distance scales. First, large-area nanosheets were transferred from the growth substrate onto a Cu TEM grid using the process described in Supporting Note 2 with a PMMA support layer, although the final H_2O rinse was omitted to ensure that the nanosheet was not accidentally removed from the grid after transfer. The grid-supported nanosheets, which possess thicknesses between 35 nm and 60 nm, conformed to the grid walls as shown in Figure S2a, leading to some tearing and bending. Consequently, the TEM image in Figure S2b shows a high density of bend contours. However, the region shown in Figure S2c exhibits a more modest density of bend contours and was chosen for selected area diffraction. A large aperture of 16 μm was used to sample a large area of the nanosheet, resulting in the SAED pattern shown in Figure S2d. This pattern indicates the α-MoO_3 crystal structure with a single orientation, finding no evidence of micro- or nano-scale grains and suggesting that the as-grown nanosheets are single crystalline. The nonuniform intensity of the diffraction spots is likely due to the slight bending across the probed area due to the TEM prep.

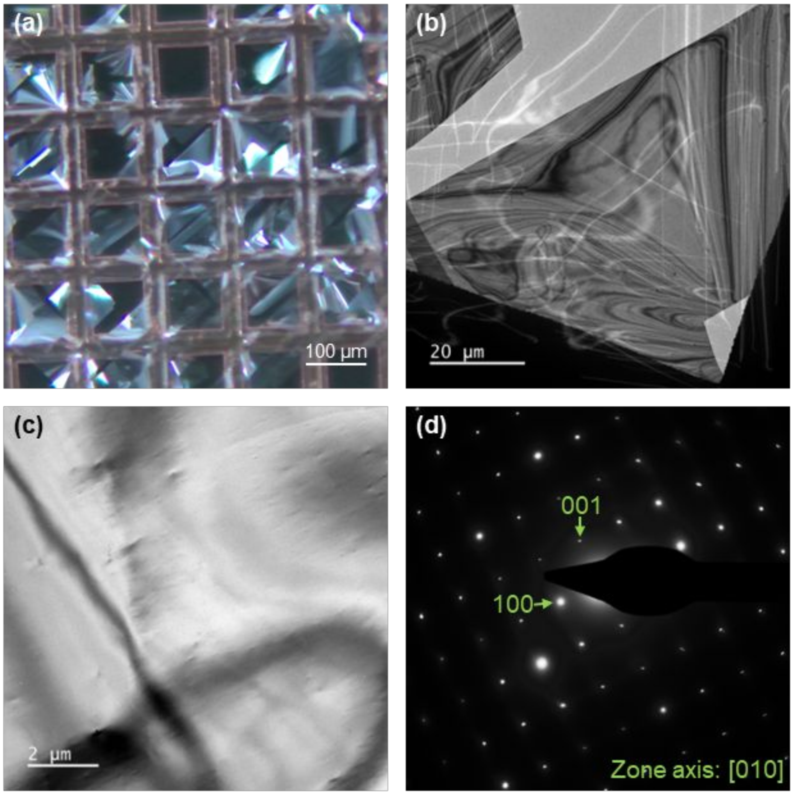

Figure S2. Plan-view TEM characterization. **(a)** Optical micrograph of α-MoO_3 nanosheet after transfer onto a Cu grid. **(b)** Low-magnification TEM image showing bend contours. **(c)** BF-TEM image and **(d)** corresponding SAED pattern of a relatively flat region collected using a 16 μm diameter selected area aperture.

We also performed electron backscatter diffraction (EBSD) on a SA-CVD nanosheet, which is shown in Figure S3a and was chosen due to its thickness (*d* = 480 nm) to maximize the EBSD signal intensity. The out-of-plane inverse pole figure (IPF) map in Figure S3b shows uniform α-MoO_3 with the *b*-axis oriented out of plane. The in-plane IPF map in Figure S3c shows a nearly uniform red color, indicating a single in-plane orientation without visible grain boundaries across the entire 0.8 × 3 mm crystal. The thin lines of different coloration marked by the white arrows are buckles which can hamper indexing of the electron backscattered pattern and occurred during sample shipment and preparation for EBSD. The rectangle showing a high density of mis-indexed points near the nanosheet center results from carbon contamination that occurred while focusing with the SEM prior to collecting the IPF maps.

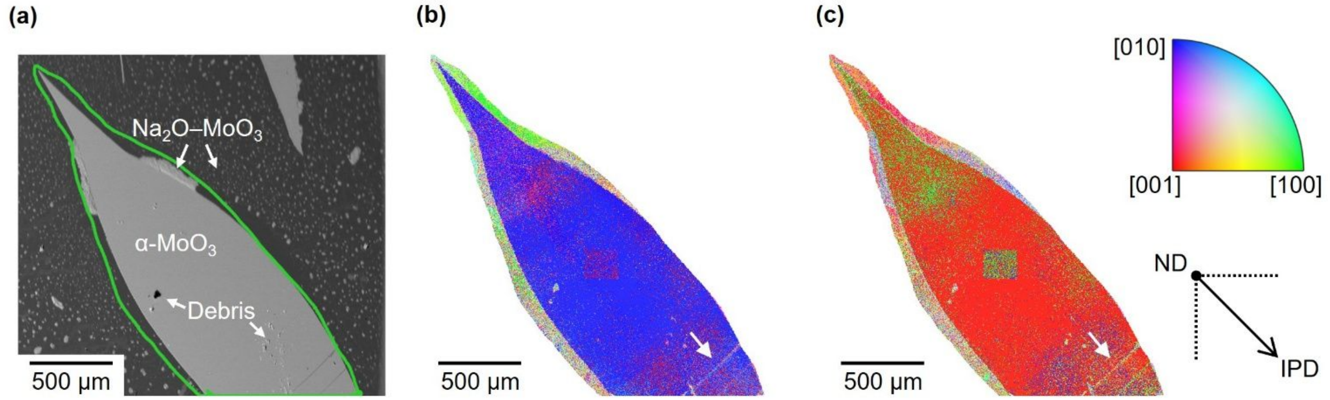

Figure S3. EBSD characterization of α-MoO_3 nanosheets. **(a)** SEM micrograph of the large 480-nm-thick nanosheet. The area measured by EBSD is outlined in green. **(b,c)** EBSD IPF maps of the nanosheet analyzed along the **(b)** normal direction (ND) and the **(c)** labeled in-plane direction (IPD).

We performed polarized light microscopy (PLM) on several α-MoO_3 nanosheets grown by the SA-CVD method, shown in Figure S4. The in-plane birefringence of the crystal structure allows determination of relative in-plane orientations by PLM. The variety of nanosheet colors indicates numerous different in-plane orientations of the nanosheets, suggesting the absence of preferred growth orientation(s). Each nanosheet is a solid color without apparent grain boundaries, further supporting the observation that the nanosheets are single-crystalline.

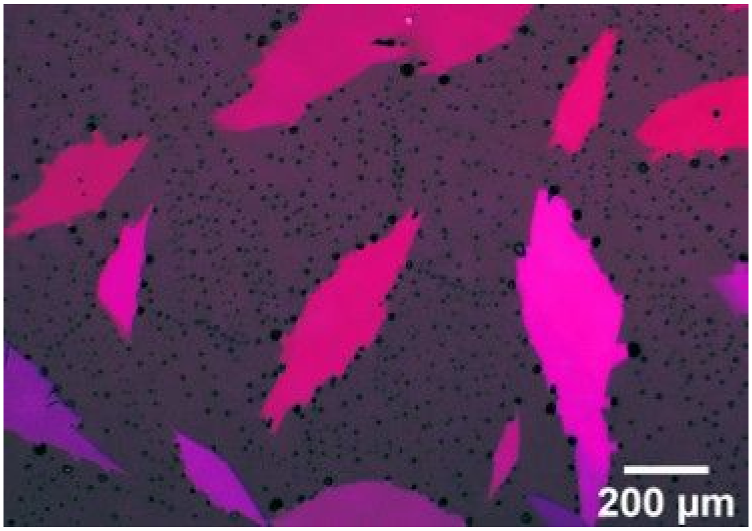

Figure S4. Polarized light micrograph of numerous α-MoO_3 single crystals of similar thicknesses (~15 nm) grown on A-plane sapphire.

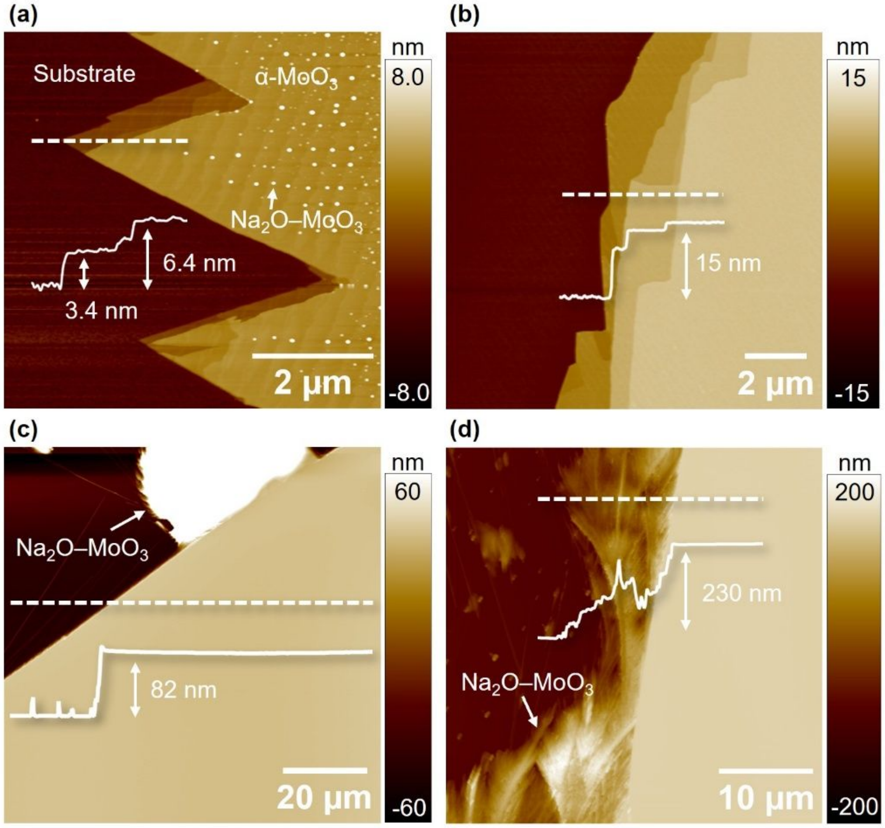

Figure S5. AFM images and line traces of α-MoO_3 nanosheets with thicknesses of **(a)** 6.4 nm, **(b)** 15 nm, **(c)** 82 nm, and **(d)** 230 nm. Solidified residual Na_2O–MoO_3 is visible as nanodroplets in (a), as a macro-scale droplet in (c), and as a front along the nanosheet edge in (d).

Supporting Note 2. Methods for wet transfer of SA-CVD nanosheets

We explored both polystyrene (PS) and poly(methyl methacrylate) (PMMA) as the polymer support layer for the wet transfer of the SA-CVD nanosheets. This process is shown in Figure S6, and is based on similar techniques which have previously been demonstrated for 2D TMD nanosheets.[1,2] A hydrophilic surface is first generated by treating the α-MoO_3-covered substrate for 20 minutes in a UV-ozone cleaner. Then, 50 μL of 10% (by weight) PS (M_w = 230,000 g/mol) in toluene (or, for PMMA, two layers of 50 μL PMMA A4 solution) is spin coated onto a UVO-treated sample at 2500 rpm for 75 s. For PS films, the sample is then dried under ambient conditions for 12 hours. The margins of the polymer coating are outlined using a razor blade to facilitate liftoff. The sample is then slowly immersed in DI H_2O, causing the α-MoO_3/polymer stack to lift off the substrate and float on the liquid surface, where it may be transferred onto an arbitrary substrate and dried on a hot plate at ~80 °C for 10 min. The transferred membrane is then immersed in toluene (or acetone for PMMA supports) for 10 min to dissolve the polymer layer and then rinsed in methanol. We note that using PS, only some of the α-MoO_3 nanosheets are removed from the original substrate; in contrast, the PMMA support layer released easily from the sapphire substrate and successfully removed all α-MoO_3 crystals, thus performing better than PS for wet transfer of the SA-CVD α-MoO_3 nanosheets. Optical micrographs of a α-MoO_3 nanosheet before and after transfer are shown in Figure S7a, showing successful transfer although some cracking or buckling may occur. By rinsing in DI H_2O after transfer, we can effectively remove the Na signal as measured *via* XPS without buckling (Figure S7b). Additionally, the Raman peaks return to their bulk positions (Figure S7c) after transfer, indicating strain relaxation. We note that the degree of cracking/buckling in the transferred nanosheets is far less catastrophic than when non-transferred nanosheets are rinsed in H_2O, which causes rapid strain relaxation.

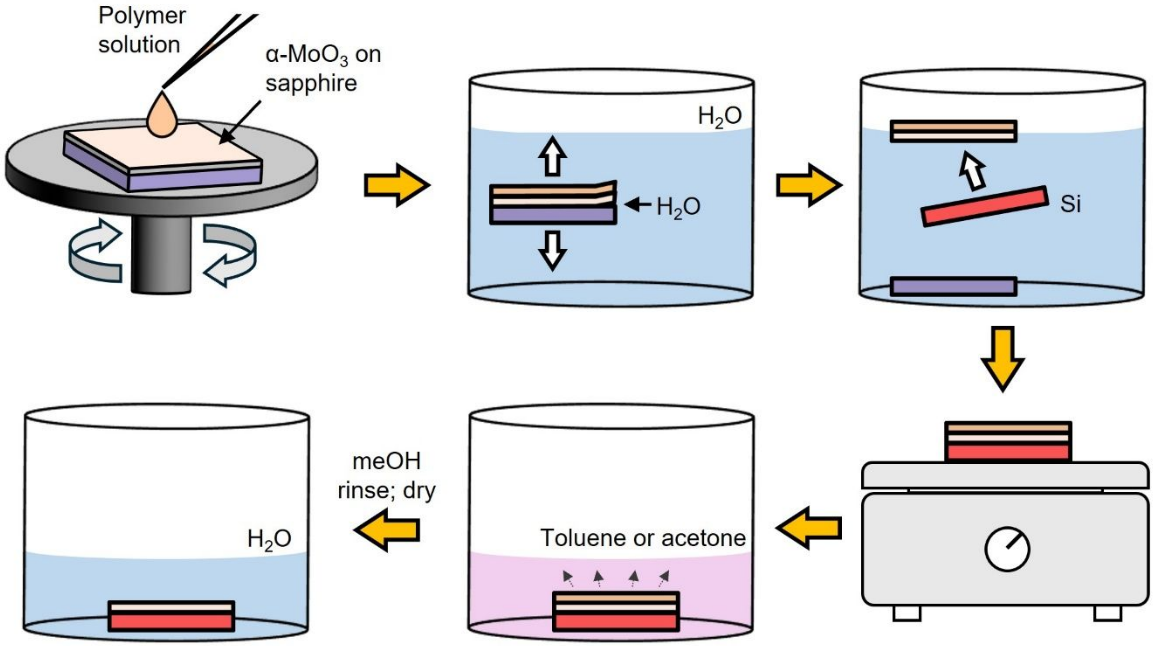

Figure S6. Schematic of the wet transfer process of α-MoO_3 nanosheets using a polystyrene support layer.

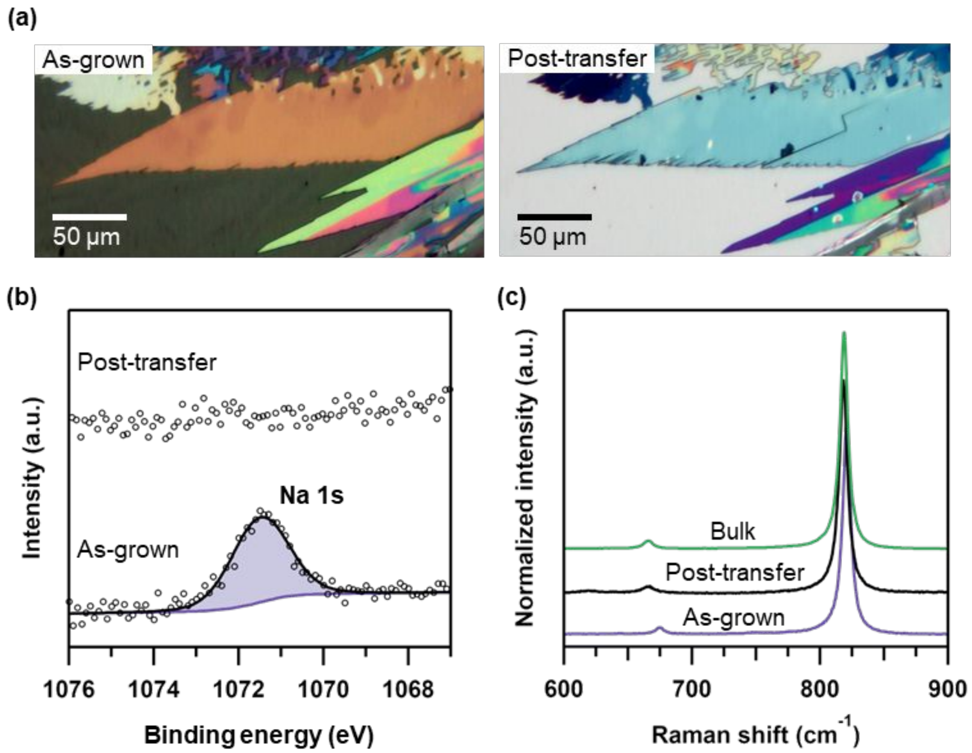

Figure S7. Effectiveness of wet transfer process. **(a)** Optical micrographs of an α-MoO_3 nanosheet before and after transfer onto a SiO_2/Si substrate. **(b)** High-resolution XPS spectra of Na 1s core levels and **(c)** Raman spectra of an as-grown and transferred nanosheet. Panel (c) also shows a Raman spectrum obtained from an unstrained bulk α-MoO_3 crystal to illustrate relaxation of the ~665 cm^{-1} peak towards bulk values following layer transfer.

Supporting Note 3. Additional interface characterization and STEM imaging

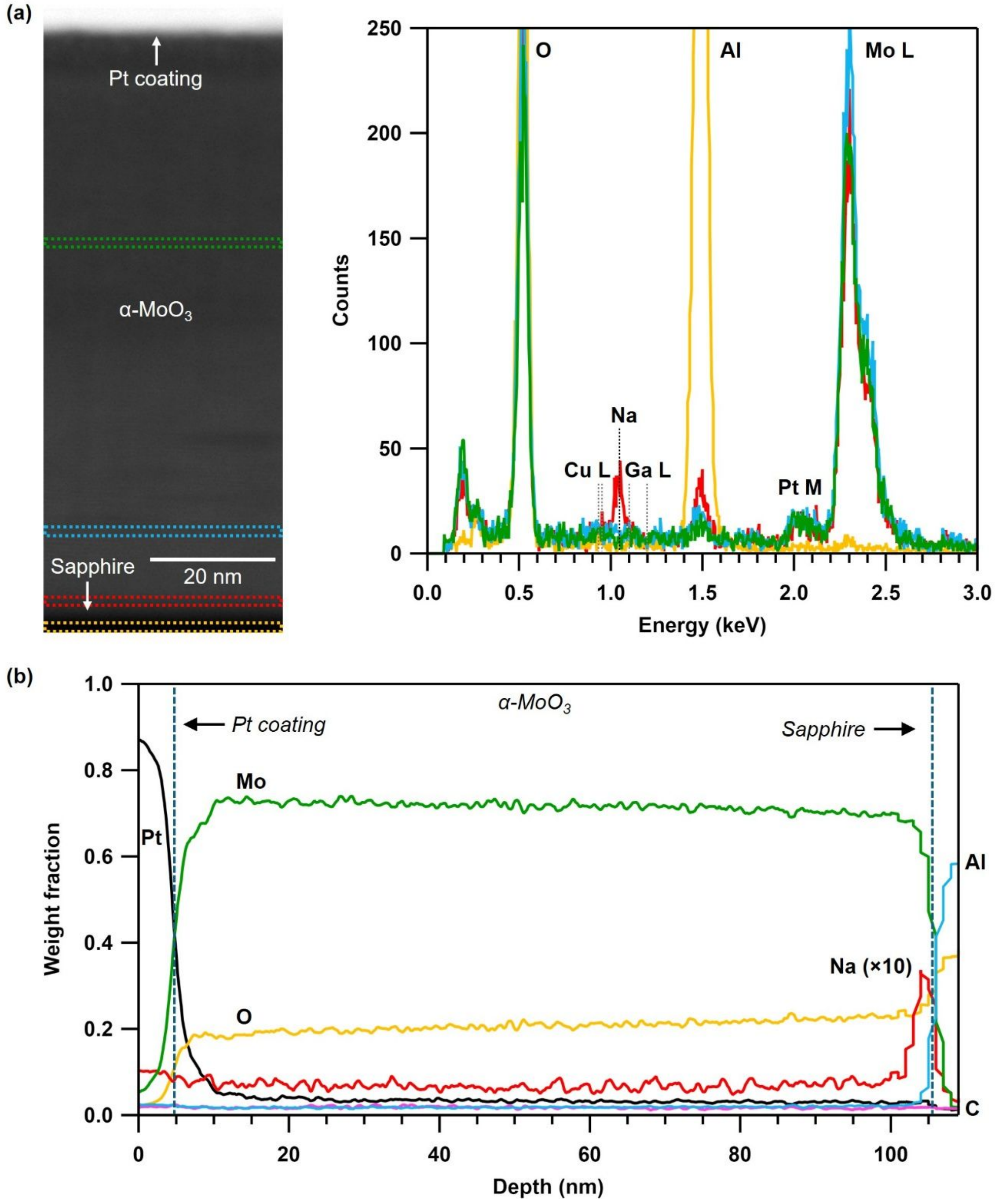

Figure S8. STEM-EDS spectra and line profiles. **(a)** EDS spectra extracted from the (yellow) sapphire substrate, (red) Na-rich interface, (blue) lower portion and (green) upper portion of the α-MoO_3 nanosheet. Background Pt signal results from the TEM environment or from re-deposition of the Pt protective layer during FIB. **(b)** EDS weight fractions along a line profile extending from the Pt topcoat to the sapphire substrate showing the Na signal is increased at the α-MoO_3/sapphire interface.

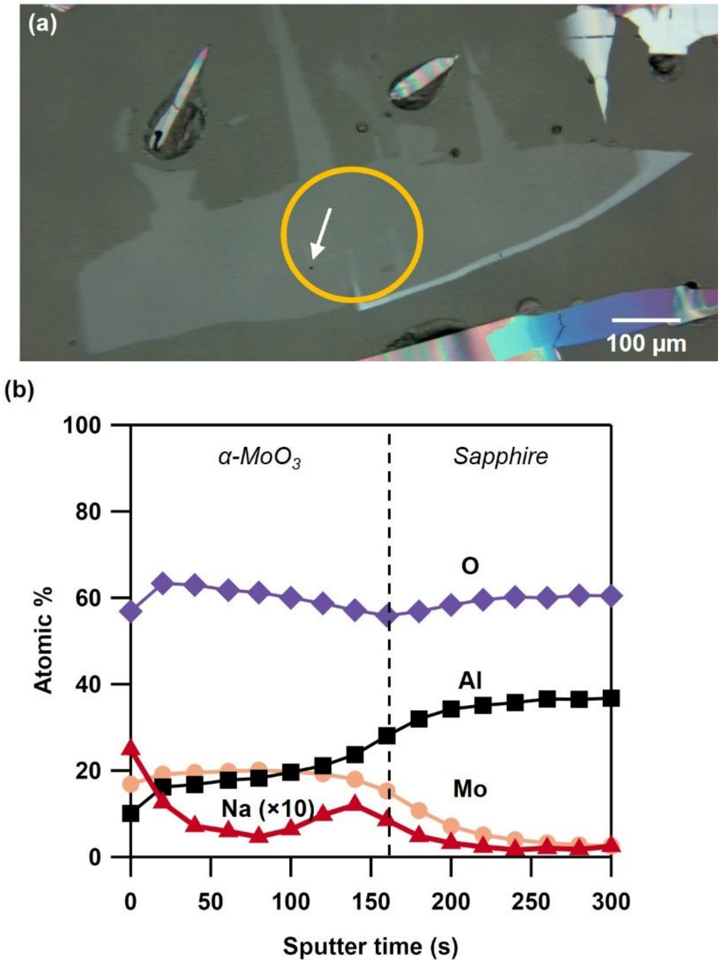

Figure S9. XPS depth profile of α-MoO_3 nanosheet. **(a)** Optical micrograph of the α-MoO_3 nanosheet with d = 15 nm that was analyzed using XPS depth profiling. The orange circle denotes the 200 µm diameter of the analysis region. The arrow indicates a residual droplet that likely caused the non-zero Na background signal during profiling. **(b)** XPS depth profile analysis showing a peak in the Na signal at the interface between the α-MoO_3 nanosheet and sapphire substrate.

The in-situ growth of unidentified crystals was observed during high-magnification STEM analysis of the α-MoO_3/sapphire interface. Initially, there is a distinct, bright contrast band between the two layers (Figure S10, Frame 1), but further dwelling on this region results in rapid growth of additional crystallites. To study this, a series of 50 frames of 2048 × 2048 pixels were acquired using 200 ns pixel dwell times over 19.51 nm × 19.51 nm regions. The images were aligned with respect to one another post-acquisition using a bandpass filter within Digital Micrograph to correct for sample drift. With an incident beam current of approximately 200 pA (Figure S10), significant contrast changes at the interface are observed across the image series, with the appearance of additional lattice planes becoming more pronounced by the last frame (Figure S10, Frame 50). Similar interfacial crystal growth was also noticed at approximately 260, 150, 100, and 50 pA beam currents utilizing identical imaging parameters. The resultant crystals demonstrated crystallographic alignment with the sapphire while distorting the surrounding α-MoO_3 (Figure S11).

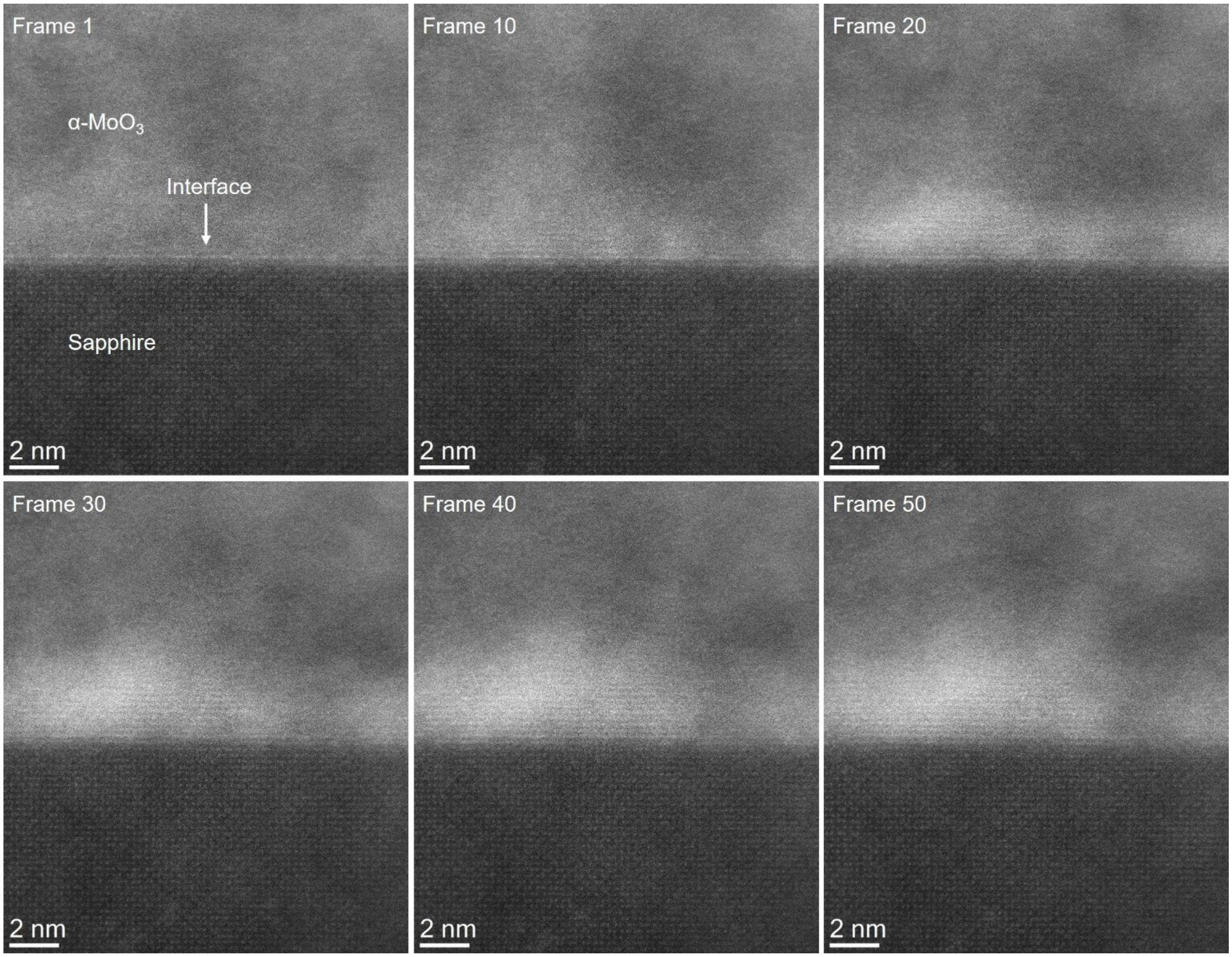

Figure S10. Timelapse STEM images under 200 pA beam current showing the progressive growth of an interfacial crystalline phase with bright contrast. The interfacial phase shares a high-symmetry zone axis with the substrate but not the α-MoO_3.

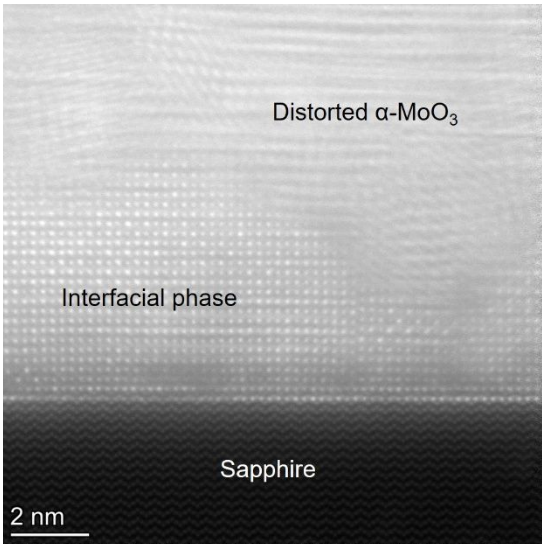

Figure S11. High-resolution STEM image showing the highly ordered interfacial phase that grows during imaging and the resulting distortion of the overlying α-MoO_3 layers.

Following sample preparation via FIB for STEM imaging, regions with darker contrast appear at the top of the cross section which exhibit reduced crystalline order compared to the lower α-MoO_3 layer (Figure S12a,b). Notably, we observed no indication of crystalline disorder of this type in any of the as-grown nanosheets through XRD, Raman spectroscopy, and other characterization techniques. Additionally, a second cross section, shown in Figure S12c, was prepared and exhibits a significantly different damage profile despite being from the same crystal as in Figure S12a,b. The second lamella was prepared with a different technique; namely, plasma FIB (PFIB) using a Xe^+ source. Therefore, the disorder apparently occurs during FIB milling and is likely not representative of the pristine nanosheets.

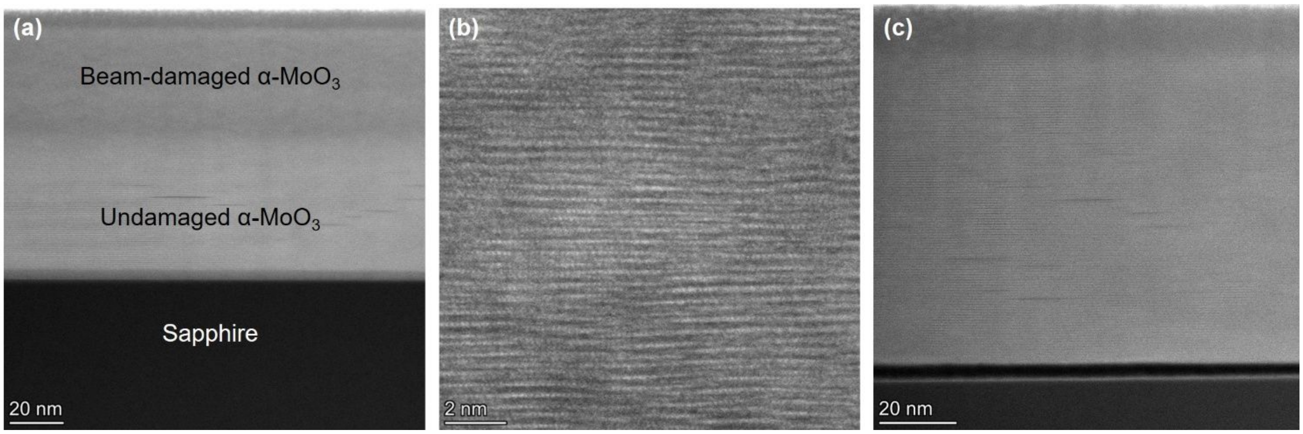

Figure S12. FIB-induced damage of α-MoO_3 layers. **(a)** Low-magnification STEM image of the entire cross-section and **(b)** high-magnification image of the damaged upper region, showing increased crystalline disorder. **(c)** STEM image of a second lamella prepared from the same crystal as (a,b) prepared using PFIB. The dark band at the bottom of the α-MoO_3 layer may be due to partial delamination.

Supporting Note 4. SEM/EDS of Na_2O–MoO_3 residual droplet

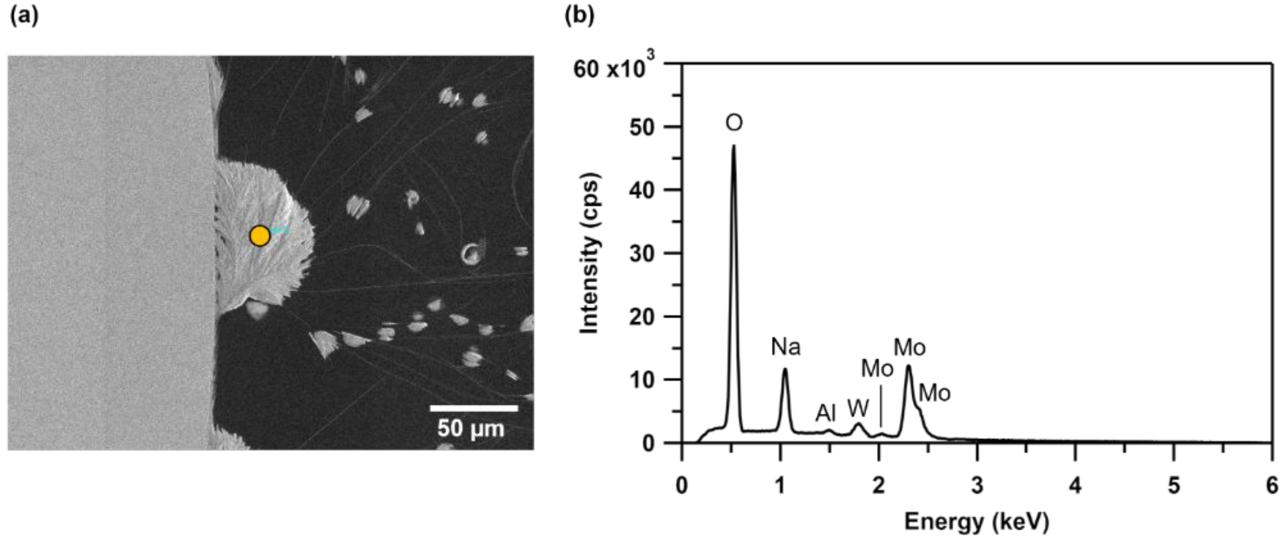

Figure S13. EDS of Na_2O–MoO_3 droplets. **(a)** SEM image of the region of interest. The orange circle marks the location analyzed in panel **(b)** representative EDS spectrum of the residual droplet. The aluminum signal results from the sapphire substrate.

Table S1. Approximate composition of Na_2O–MoO_3 droplets (with carbon excluded) averaged from 5 measurements.

Element	Atomic fraction	Weight fraction
Oxygen	0.67	0.33
Sodium	0.11	0.08
Molybdenum	0.19	0.57
Aluminum	0.26	0.02

Supporting Note 5. Temperature profile

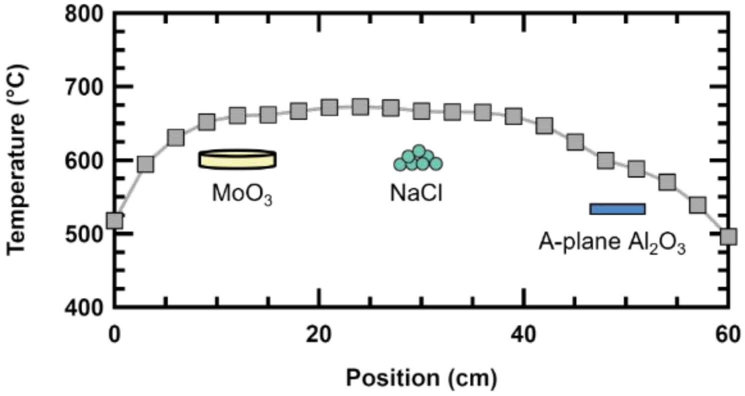

Figure S14. Horizontal temperature profile of the growth chamber. Temperature was measured using an external Type K thermocouple positioned near the axial center line of the 50-mm-OD tube. The set point temperatures were: T_{MoO_3} = 625 °C, T_{NaCl} = 630 °C, $T_{substrate}$ = 550 °C.

Supporting Note 6. SA-CVD α-MoO_3 morphology evolution

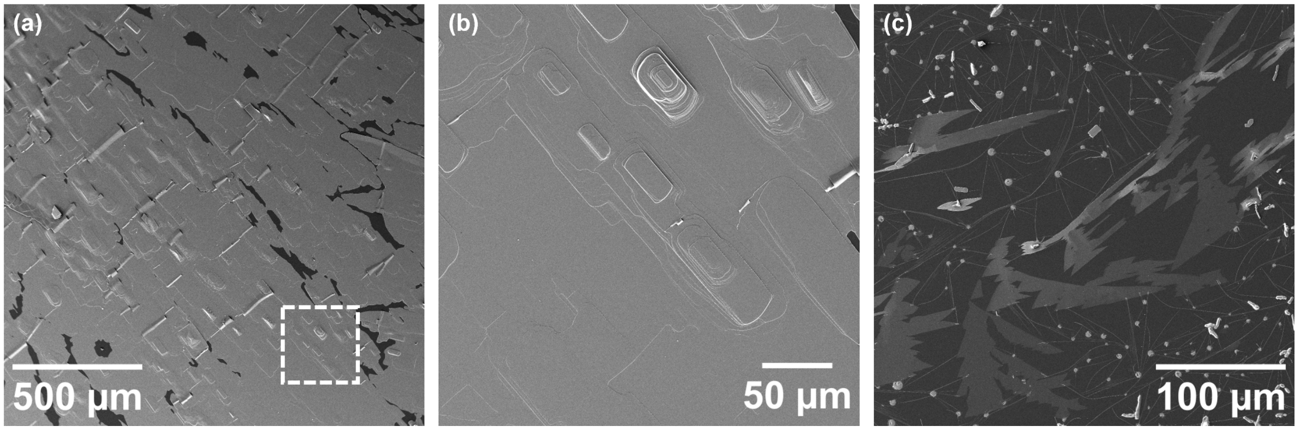

Figure S15. Additional morphologies observed at T_{MoO_3} = 645 °C. SEM micrographs of **(a,b)** mesa-covered and **(c)** dendritic morphologies present on the same sample. Image (b) corresponds to the outlined region of (a).

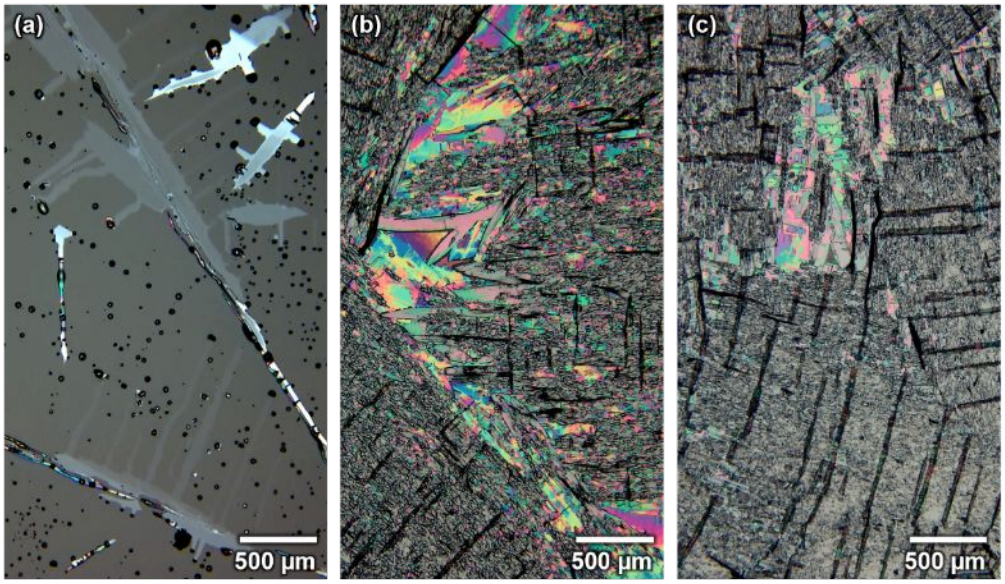

Figure S16. Optical micrographs of samples grown using a powder geometry of the α-MoO_3 sublimation source with a mass of **(a)** 45 mg, **(b)** 75 mg, and **(c)** 125 mg.

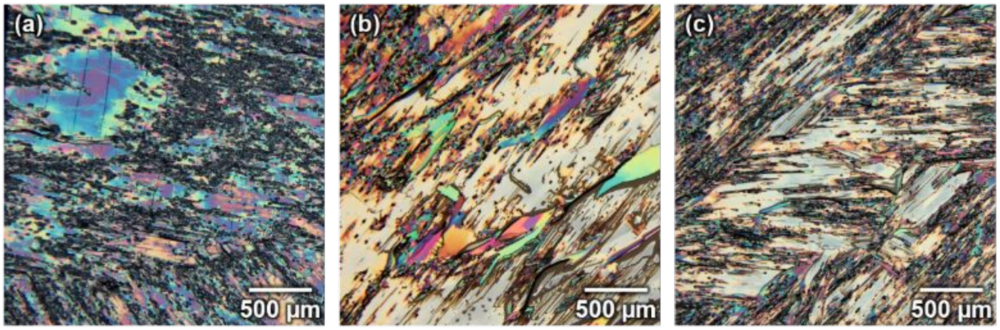

Figure S17. Sample-to-sample reproducibility. **(a-c)** Optical micrographs of samples grown in succession under identical conditions, resulting in nanosheets covered with mesas which appear dark in these images. The morphologies of the three samples are relatively similar; different coloration corresponds to nanosheet thickness, which often varies even across a single sample.

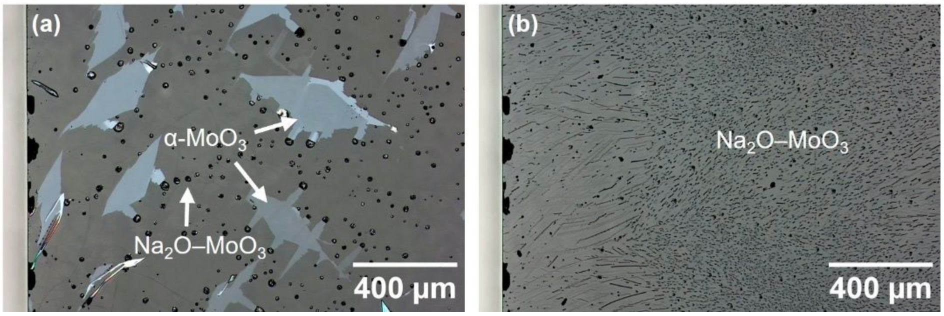

Figure S18. Thermal instability of α-MoO_3 at the growth temperature. α-MoO_3 sheets grown by the SA-CVD method **(a)** before and **(b)** after post-annealing under the growth conditions (550 °C, 350 sccm O_2 at 10 mTorr, 15 min) with no α-MoO_3 or NaCl sources inserted. Following the annealing treatment, the α-MoO_3 sheets have either evaporated or been consumed by the Na_2O–MoO_3 low-melting-temperature phase.

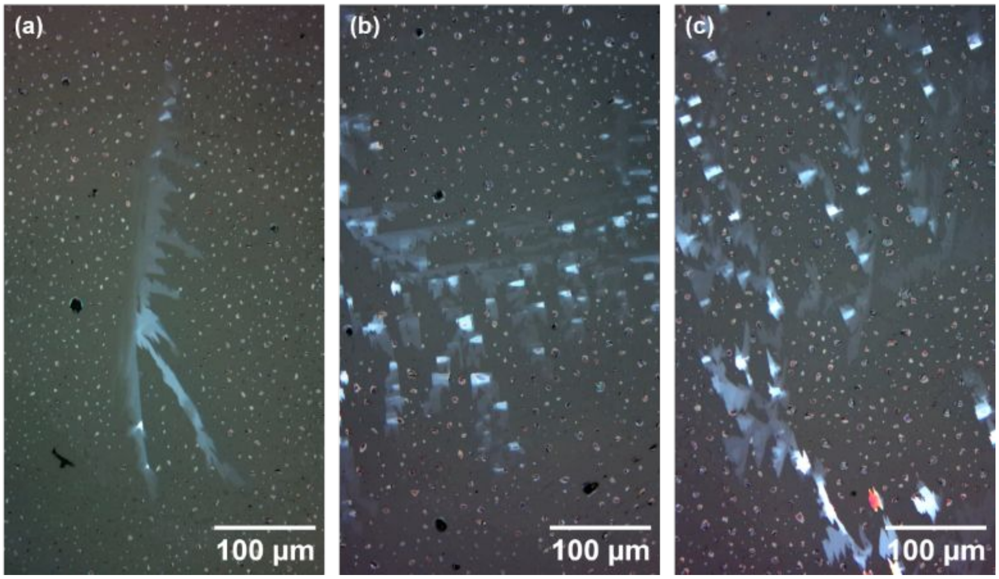

Figure S19. Duration-insensitive SA-CVD growth under MoO_3-scarce conditions. α-MoO_3 nanosheets grown with dendritic morphologies using a low T_{MoO_3} for durations of **(a)** 7.5 min, **(b)** 15 min, and **(c)** 30 min. The length and width of the dendrites ultimately do not increase with growth duration, indicating that the decomposition rate rivals the growth rate to prevent large-area growth.

Supporting Note 7. α-MoO_3 growth on different substrates

We perform SA-CVD growth simultaneously on several different substrates and show SEM images of the growth on C-plane sapphire, Si (100), and muscovite mica in Figure S20. Evidently, the choice of A-plane sapphire substrates is critical to achieve large-area, uniform α-MoO_3 sheets under our growth conditions. Growth on C-plane sapphire (Figure S20a,b) results in significantly smaller nanosheet dimensions (< 50 μm) compared to A-plane sapphire. The residual droplets present on the C-plane sapphire surface are also significantly smaller and more numerous. We suspect that the differing surface energies of the substrates affect the motion of the liquid droplets, ultimately altering the nanosheet morphology. On Si, no large crystals can be grown; instead, we find only inclined nanoplates protruding out of the substrate, potentially due to chemical reactions between the alkali salt and substrate (Figure S20c). Furthermore, MoO_3 has been shown to spontaneously disperse more favorably on sapphire than SiO_2 surfaces, which may also affect this growth mode by modifying interfacial interactions.[3–5]

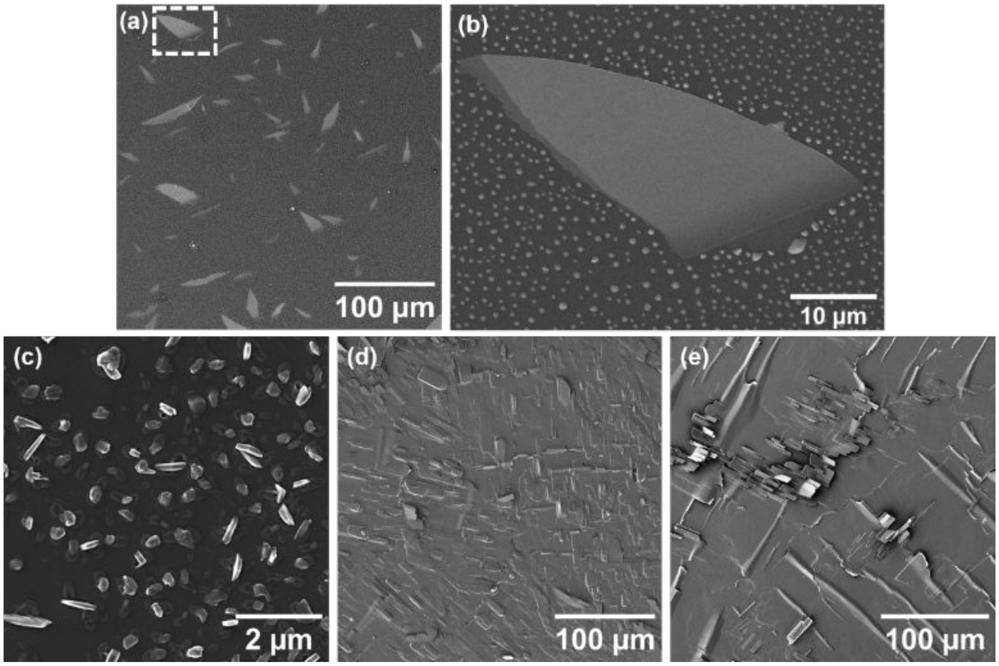

Figure S20. SEM images of α-MoO_3 grown on different substrates. SEM micrographs of SA-CVD growth on **(a,b)** C-plane sapphire, **(c)** Si (100), and **(d)** muscovite mica substrates. Image (b) is a close-up view of the outlined region in image (a). **(e)** α-MoO_3 growth on muscovite mica without the NaCl source inserted, exhibiting nominally similar morphology to (d).

On mica, large-area, albeit rough, α-MoO_3 crystals can be grown with or without the use of a NaCl source as shown in Figure S20d,e. XPS analysis reveals that the surface of the as-received mica substrate contains both K and Na species (Figure S21a,b). After PVT growth of α-MoO_3 with no NaCl added, XPS was performed again and the results are summarized in Figure S21c. The amount of Na remains unchanged (or increases) despite the exposed surface area of the substrate, estimated by Si and Al quantities, being significantly reduced after α-MoO_3 growth. This indicates that Na atoms initially present in the substrate participate in the growth and "float" on the surface of the growing nanosheet. Based on the mesa-dense morphology and absence of residue from a liquid phase, we suspect that growth on mica occurs through a VS or VSS alkali metal compound-assisted growth mode. However, even when we employ a NaCl evaporative source, we are unable to produce smooth and large-area α-MoO_3 morphologies on mica (Figure S20d). Additionally, the mica substrate is chemically reactive with MoO_3; after PVT growth with no NaCl source, multiple reaction products are identified in the XRD pattern shown in Figure S21d. These products do not form when mica substrates are annealed in the absence of MoO_3 under the growth conditions ($T_{substrate}$ = 550 °C, 350 sccm O_2 at 10 mTorr for 15 min).

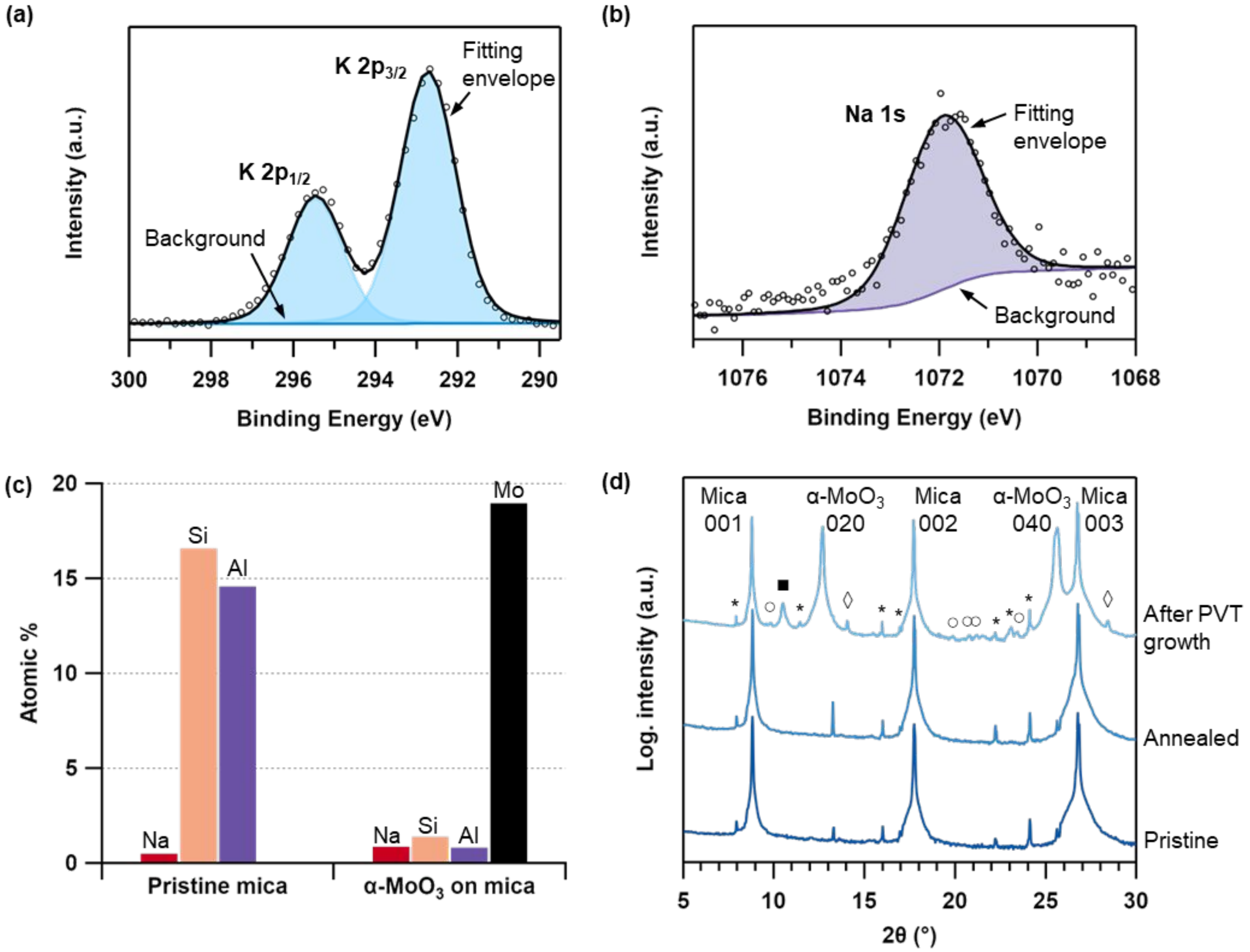

Figure S21. α-MoO_3 growth on muscovite mica without external NaCl. **(a-b)** XPS scans of K 2p and Na 1s core level spectra of the as-received muscovite mica surface, indicating presence of both alkali species prior to growth. **(c)** Relative atomic percentages of Na, Si, Al, and Mo species of the sample surface before and after α-MoO_3 growth. The amount of Na relative to the uncovered substrate area, estimated by Si and Al signal, is significantly increased after growth. **(d)** Offset XRD θ–2θ scans of muscovite mica in its pristine state, after annealing under the growth conditions without MoO_3 or NaCl sources loaded, and after α-MoO_3 PVT growth without a NaCl source. * = peaks arising from secondary and λ/2 wavelengths, ■ = cordierite ($Mg_2Al_4Si_5O_{18}$), ◊ = $Al_2(MoO_4)_3$, ○ = other phases. Phase attribution is not definite due to the limited number of peaks of the highly textured phases.

Supporting Note 8. Two-step SA-CVD growth of α-MoO_3 on A-plane sapphire

We also explored a broad range of experimental parameters in attempts to develop a two-step SA-CVD process for α-MoO_3, with a representative process described here and shown schematically in Figure S22a. First, the A-plane sapphire substrate is spin washed with de-ionized H_2O, isopropyl alcohol, and methanol followed by cleaning in a UV-ozone generator for 20 minutes to prepare a hydrophilic surface for spin coating. Then, the alkali precursor is applied by spin coating 20 µL of 1.5 mM aqueous NaOH solution at 5000 rpm for 40 s. The prepared substrate is then loaded into the three-zone tube furnace for α-MoO_3 growth with 15 mg of α-MoO_3 powder loaded into the hot zone. Zones 1, 2, and 3 are heated to 720 °C, 500 °C, and 400 °C, respectively, and growth initiated by flowing 500 sccm O_2 at 12.5 mTorr for 2 min.

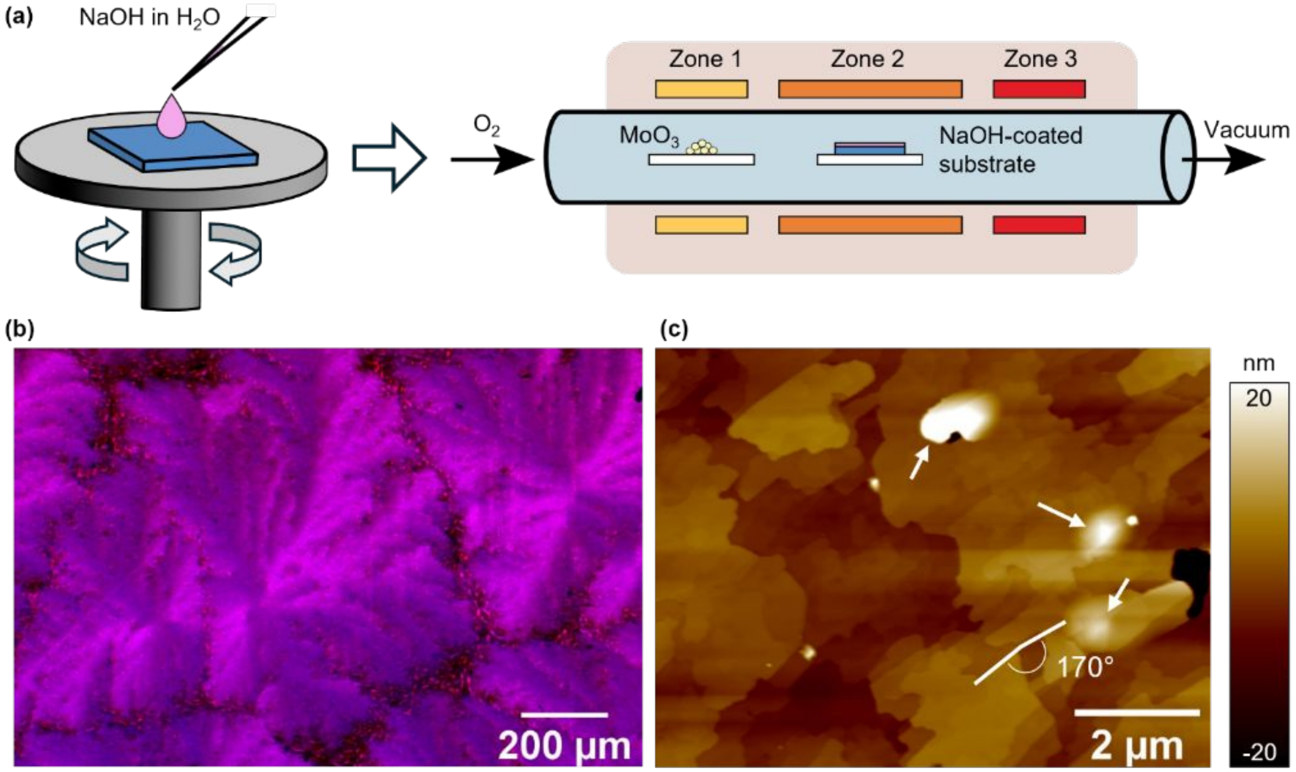

Figure S22. Two-step growth of non-crystallographically branched α-MoO_3 using spin-coated NaOH precursors. **(a)** Schematic of the two-step growth process. **(b)** Polarized light microscopy of a typical morphology resulting from the two-step growth process. **(c)** AFM height image of the same sample, displaying a disordered topography with low-angle rotations visible. The raised regions marked by arrows likely delineate residual Na_2O–MoO_3 droplets which remained trapped underneath the growing nanosheet.

Following the two-step SA-CVD growth, irregular α-MoO_3 islands are found on the sample surface. As shown in the PLM image in Figure S22b, each island is composed of numerous dendritic crystallites which exhibit different in-plane orientations, following a twisting pattern. These slight rotations between adjacent domains can be observed in the AFM height image in Figure S22c; additionally, the topography is relatively rough. This morphology resembles non-crystallographic branching, a growth outcome where dendritic crystallites progressively rotate as growth proceeds.[6,7] Inorganic materials may follow this growth mode when dependent on solid-state phase transformations for growth.[7,8] In our case, the two-step growth method may induce a VSS mechanism through the spin-coated alkali precursor. Although some liquid may form, as indicated by the potential residual droplets observed in Figure S22c, it appears insufficient to accomplish high-quality single-crystal growth through the self-expanding VLS mechanism that the single-step SA-CVD method enables.

Supporting Note 9. Additional s-SNOM results

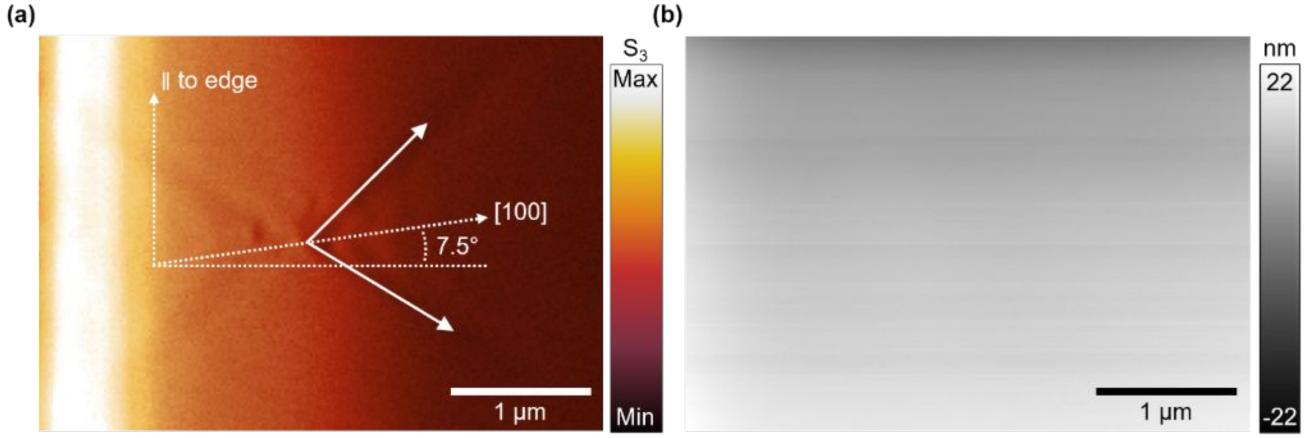

Figure S23. Misorientation of the α-MoO_3 edge and evidence of a non-topographic HPhP point launching site. **(a)** S_3 amplitude and **(b)** topographic height image of the point launching site. The angular midpoint of the point-launched fringes, which corresponds to α-MoO_3 [100], is 7.5° misaligned from the line perpendicular to the crystal edge.

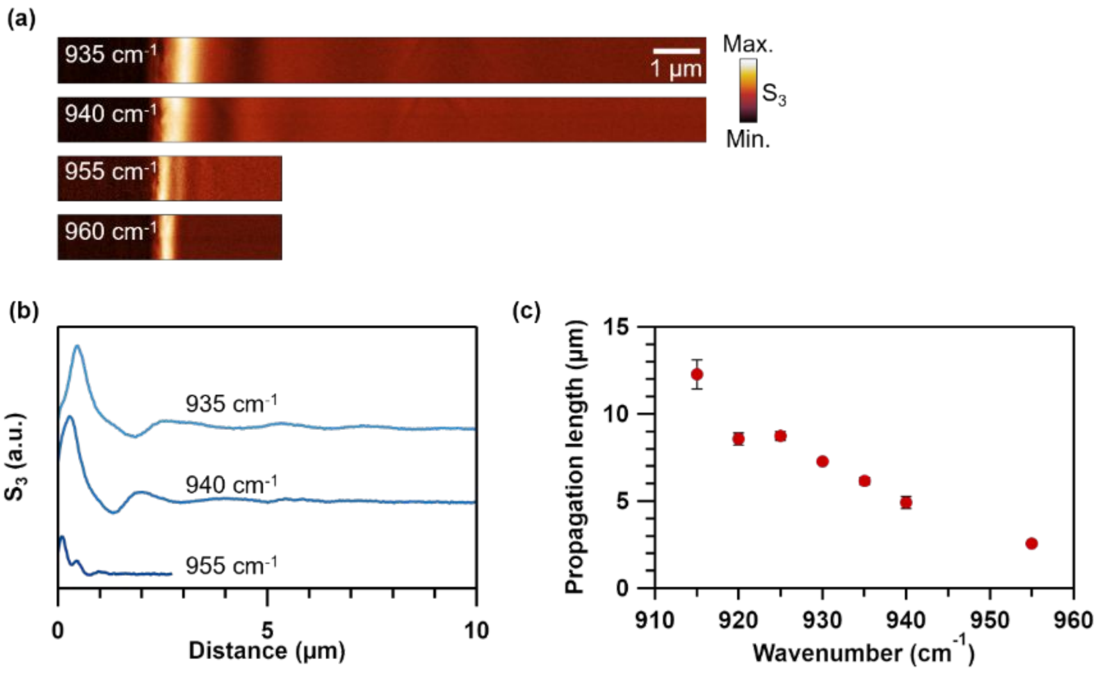

Figure S24. HPhP launching at additional frequencies. **(a)** s-SNOM S_3 maps and **(b)** extracted line profiles at higher frequencies. No line profile was extracted for the 960 cm^{-1} measurement due to rapid attenuation of the intensity fringes. **(c)** HPhP propagation lengths across the measured frequency range.

References

(1) Schneider, G. F.; Calado, V. E.; Zandbergen, H.; Vandersypen, L. M. K.; Dekker, C. Wedging Transfer of Nanostructures. *Nano Lett.* **2010**, *10*, 1912–1916. https://doi.org/10.1021/nl1008037.

(2) Elías, A. L.; Perea-López, N.; Castro-Beltrán, A.; Berkdemir, A.; Lv, R.; Feng, S.; Long, A. D.; Hayashi, T.; Kim, Y. A.; Endo, M.; Gutiérrez, H. R.; Pradhan, N. R.; Balicas, L.; Mallouk, T. E.; López-Urías, F.; Terrones, H.; Terrones, M. Controlled Synthesis and Transfer of Large-Area WS_2 Sheets: From Single Layer to Few Layers. *ACS Nano* **2013**, *7*, 5235–5242. https://doi.org/10.1021/nn400971k.

(3) Leyrer, J.; Mey, D.; Knözinger, H. Spreading Behavior of Molybdenum Trioxide on Alumina and Silica: A Raman Microscopy Study. *Journal of Catalysis* **1990**, *124*, 349–356. https://doi.org/10.1016/0021-9517(90)90183-K.

(4) El-Shobaky, G. A.; Fagal, G. A.; Hassan, N. A. Solid–Solid Interactions in Pure and Na_2O-Doped MoO_3/Al_2O_3 System. *Thermochimica Acta* **1998**, *311*, 205–211. https://doi.org/10.1016/S0040-6031(97)00410-3.

(5) Braun, S.; Appel, L. G.; Camorim, V. L.; Schmal, M. Thermal Spreading of MoO_3 onto Silica Supports. *J. Phys. Chem. B* **2000**, *104*, 6584–6590. https://doi.org/10.1021/jp000287m.

(6) Shtukenberg, A. G.; Punin, Y. O.; Gunn, E.; Kahr, B. Spherulites. *Chemical Reviews* **2012**, *112*, 1805–1838. https://doi.org/10.1021/cr200297f.

(7) Lutjes, N. R.; Zhou, S.; Antoja-Lleonart, J.; Noheda, B.; Ocelík, V. Spherulitic and Rotational Crystal Growth of Quartz Thin Films. *Sci Rep* **2021**, *11*, 14888. https://doi.org/10.1038/s41598-021-94147-y.

(8) Zhou, S.; Antoja-Lleonart, J.; Nukala, P.; Ocelík, V.; Lutjes, N. R.; Noheda, B. Crystallization of GeO_2 Thin Films into α-Quartz: From Spherulites to Single Crystals. *Acta Materialia* **2021**, *215*, 117069. https://doi.org/10.1016/j.actamat.2021.117069.
